# A customized strategy to design intercalation-type Li-free cathodes for all-solid-state batteries

**DOI:** 10.1093/nsr/nwad010

**Published:** 2023-01-10

**Authors:** Da Wang, Jia Yu, Xiaobin Yin, Sen Shao, Qianqian Li, Yanchao Wang, Maxim Avdeev, Liquan Chen, Siqi Shi

**Affiliations:** School of Materials Science and Engineering, Shanghai University, Shanghai 200444, China; Zhejiang Laboratory, Hangzhou 311100, China; Materials Genome Institute, Shanghai University, Shanghai 200444, China; School of Materials Science and Engineering, Shanghai University, Shanghai 200444, China; State Key Laboratory of Superhard Materials & International Center for Computational Method and Software, Jilin University, Changchun 130012, China; Materials Genome Institute, Shanghai University, Shanghai 200444, China; State Key Laboratory of Superhard Materials & International Center for Computational Method and Software, Jilin University, Changchun 130012, China; Australian Nuclear Science and Technology Organisation, Kirrawee DC, NSW 2232, Australia; School of Chemistry, University of Sydney, Sydney 2006, Australia; Institute of Physics, Chinese Academy of Sciences, Beijing 100190, China; School of Materials Science and Engineering, Shanghai University, Shanghai 200444, China; Materials Genome Institute, Shanghai University, Shanghai 200444, China; Zhejiang Laboratory, Hangzhou 311100, China

**Keywords:** intercalation-type Li-free cathodes, all-solid-state Li-metal batteries, energy density, p-type alloying strategy, ligand-field descriptors

## Abstract

Pairing Li-free transition-metal-based cathodes (MX) with Li-metal anodes is an emerging trend to overcome the energy-density limitation of current rechargeable Li-ion technology. However, the development of practical Li-free MX cathodes is plagued by the existing notion of low voltage due to the long-term overlooked voltage-tuning/phase-stability competition. Here, we propose a p-type alloying strategy involving three voltage/phase-evolution stages, of which each of the varying trends are quantitated by two improved ligand-field descriptors to balance the above contradiction. Following this, an intercalation-type 2H-V_1.75_Cr_0.25_S_4_ cathode tuned from layered MX_2_ family is successfully designed, which possesses an energy density of 554.3 Wh kg^−1^ at the electrode level accompanied by interfacial compatibility with sulfide solid-state electrolyte. The proposal of this class of materials is expected to break free from scarce or high-cost transition-metal (e.g. Co and Ni) reliance in current commercial cathodes. Our experiments further confirm the voltage and energy-density gains of 2H-V_1.75_Cr_0.25_S_4_. This strategy is not limited to specific Li-free cathodes and offers a solution to achieve high voltage and phase stability simultaneously.

## Teaser

A critical voltage-tuning/phase-stability competition balance of Li-free cathodes for all-solid-state Li-metal batteries is achieved by the customized p-type alloying strategy, and a conceptual 2H-V_1.75_Cr_0.25_S_4_ cathode with record initial-voltage and energy density in intercalation-type Li-free cathode field is designed.

## INTRODUCTION

The remarkable advances in battery chemistries beyond Li-ions in the past decades have revived the pursuit of the ‘Holy Grail’, i.e. Li-metal anodes for rechargeable batteries [1,2]. As a result, a Li-free cathode/solid-state electrolyte/Li system with higher energy density and safety is again one of the strongest research trends of next-generation Li-based batteries [3–5]. Intercalation-type Li-free cathodes in general enable more reversibility since the topotactic Li^+^-(*de*)intercalation avoids drastic changes in both micro/macro structures and physical properties [6]. As early as the 1970s, TiS_2_ was first demonstrated as a typical intercalation-type cathode for rechargeable batteries [7], which was successfully applied in a solid-state battery afterwards [8]. Subsequently, a number of transition-metal sulfides (MS) with higher S-3p energy levels were investigated due to their high safety and (*de*)intercalation reversibility (Supplementary Fig. S1). For example, Mai *et al.* [9] have reported vanadium-based disulfide (VS_2_) sheets as excellent alkali–metal-ion storage electrodes. Based on *in situ* X-ray diffraction (XRD), a key two-phase reaction ion storage mechanism (VS_2_ to Li_0.33_VS_2_) followed by a solid-solution reaction (Li_0.33_VS_2_ to LiVS_2_) was revealed, which is of great importance and has inspired the development of intercalation-type MS cathodes. Besides, Li *et al*. [10] reported densely arranged VS_2_ sheets coated by TiS_2_ with lattice distortion and high crystallinity, in which TiS_2_ surface coating effectively preserved the delamination and break-up of VS_2_ sheets during lithiation–delithiation. These findings provide new opportunities for the rational design of MS materials for building high-performance Li-ion batteries. Besides, since cathode materials suitable for large-scale energy-storage applications (e.g. electric vehicles) are all reliant on scarce and high-cost Co or Ni to some degree [11–13], the advantages of these Co/Ni-excluded sulfides as alternative materials are quite attractive. Most importantly, such Li-free sulfide cathodes are expected to solve the long-standing open problem of large interfacial resistance caused by the large O–S electronegativity difference between the most widely studied sulfide solid-state electrolytes and the traditional Li-containing oxide cathodes [14,15].

However, the intrinsic low average voltage (≤2 V) accompanied by low energy density (≤450 Wh kg^−1^) at the electrode level severely hinders the applications of MS materials as cathodes (Supplementary Fig. S1) and it is highly desirable to enhance their voltage. The commonly used strategies on traditional cathodes are essentially motivated by changing the ionic/covalent nature of metal–ligand bonds, which are rather complicated to control, and more importantly, the approach could not so far achieve a direct and significant increase in cathode voltage (Supplementary Section S1 and Supplementary Fig. S2). Thus, a practical question has remained for decades: Can the energy densities of intercalation-type Li-free MS materials be optimized to a level comparable to those of Li-containing oxide cathodes (>550 Wh kg^−1^)?

It is generally known that the redox energy level of a Li-free MS cathode should be as low as possible with respect to that of the Li-metal anode to achieve a large cell-reaction energy ($\Delta {\rm{G}}$), which is the prerequisite to an ideal Li^+^-intercalation voltage [16]. $\Delta {\rm{G}}$ can be separated in the way of a Born–Haber cycle: (i) ${\rm{Li}} = {\rm{L}}{{\rm{i}}}^ + + {e}^ - $ ($\Delta {{\rm{G}}}_{\rm{i}}$), (ii) ${\rm{MS}} + {e}^ - = {\rm{M}}{{\rm{S}}}^ - {\rm{\ }}( {\Delta {{\rm{G}}}_{{\rm{ii}}}} )$, (iii) ${\rm{L}}{{\rm{i}}}^ + + {\rm{M}}{{\rm{S}}}^ - = {\rm{LiM}}{{\rm{S}}}^{\rm{*}}{\rm{\ }}(\Delta {{\rm{G}}}_{{\rm{iii}}}$, * denotes Li^+^-intercalation structure$){\rm{\ }}$[17]. It can be seen that $\Delta {{\rm{G}}}_{\rm{i}}{\rm{\ }}$represents the inherent property of lithium, while $\Delta {{\rm{G}}}_{{\rm{iii}}}$ is difficult to control because it is dominated by the Li^+^ coordination energies and the structural distortion caused by the Li^+^ coulombic repulsion. Crucially, $\Delta {{\rm{G}}}_{{\rm{ii}}}$ corresponds to the work function of the MS host and is closely related to the energy gain of bringing a Li-2s electron to its Fermi level. Yet the specific design rules based on work functions and their influence on battery performance are relatively underexplored, which provides considerable optimization space. This fact inspires us to explore a new strategy to significantly enhance the voltage and energy density of Li-free MS cathodes by tuning their electronic structures.

In this work, we identify a crucial voltage-tuning/phase-stability competition as a key to developing Li-free cathodes with enhanced voltages. This is achieved by a p-type alloying strategy involving three essential stages, i.e. molecular-orbital transformation, ligand-field transition and M-valence-state change. Each of them is quantitatively characterized by two ligand-field descriptors, which enables controlling the balance between voltage and phase stability. As a proof of this strategy, a new family of high-voltage V–Cr–S cathodes tuned from Group-VIB/VB MX_2_ have been successfully designed. Among them, the optimal 2H-V_1.75_Cr_0.25_S_4_ cathode has a high initial Li^+^-intercalation voltage of 2.767 V and, ultimately, a record-breaking theoretical energy density of 554.3 Wh kg^−1^ among the intercalation-type Li-free electrodes. In addition, it provides a smooth Li^+^ interfacial migration path with a representative sulfide solid-state electrolyte, thus enabling lower interfacial resistance than traditional oxide electrodes. Subsequent experimental results confirm its advantage operation voltage and energy density, demonstrating the effectiveness of a p-type alloying strategy in designing intercalation-type Li-free cathodes customized for all-solid-state Li-metal batteries.

## RESULTS

### A p-type alloying strategy for enhancing the voltages of MX_2_ cathodes

**Figure 1.**
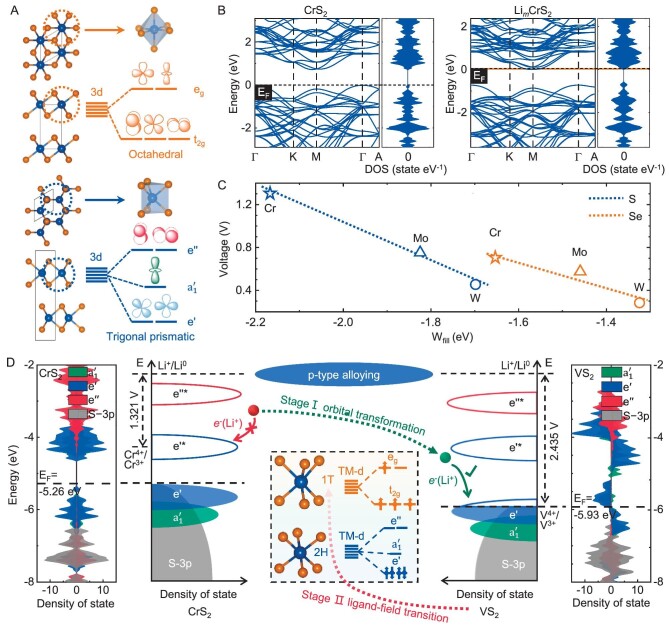
Voltage enhancement of MX_2_ cathodes by p-type alloying strategy. (A) Top and front view of MX_2_ structures with octahedral (1T) and trigonal prismatic (2H) ligand fields. (B) Electronic band structures and DOS of CrS_2_ and Li*_m_*CrS_2_ (*m* = 0.0625), respectively. (C) Average voltage as a function of W_fill_ for 2H-LiMX_2_ (M = Cr, Mo, W; X = S, Se). The dotted lines show that the MX_2_ cathode voltages gradually decrease with element periods. (D) Schematic of p-type alloying strategy. Taking 2H-CrS_2_/VS_2_ as an example, two voltage-evolution stages that are involved in the p-type alloying strategy, i.e. molecular-orbital transformation (Stage I) and ligand-field transition (Stage II), are illustrated.

We start by considering the structural and the electronic hybridization characteristics of MX_2_ (X = S, Se) electrodes to explore the origination of their low Li^+^-intercalation voltage. There have been >740 reports in the past decade on improving the capacity, electronic conductivity and cycling performance of MX_2_ as hosts for rechargeable batteries (Supplementary Fig. S3). MX_2_ mainly crystallizes in 2H (space group $P{6}_3/mmc$, #194) and 1T (space group $P\bar{3}m1$, #164) polymorphs, in which the M-3d orbitals are split into [${\rm{e^{\prime}}}$ (${{\rm{d}}}_{{\rm{xy}}}$, ${{\rm{d}}}_{{{\rm{x}}}^2 - {{\rm{y}}}^2}$), ${\rm{a}}_1^{\rm{^{\prime}}}$ (${{\rm{d}}}_{{{\rm{z}}}^2}$) and ${\rm{e^{\prime\prime}}}$ (${{\rm{d}}}_{{\rm{xz}},{\rm{\ yz}}}$)] and [${{\rm{e}}}_{\rm{g}}{\rm{\ }}({{\rm{d}}}_{{{\rm{z}}}^2}$,${\rm{\ }}{{\rm{d}}}_{{{\rm{x}}}^2 - {{\rm{y}}}^2}$) and t_2g_ (${{\rm{d}}}_{{\rm{xy}},{\rm{ xz}},{\rm{ yz}}}$)], respectively (Fig. 1A) [18]. From the relative total energies between the 1T- and 2H-phases shown in Supplementary Fig. S4 and Supplementary Table S1, the 2H-phases have lower energies especially for Group-VIB MX_2_ [19].

It can be inferred from the linear relation between the average Li^+^-intercalation voltage and ${{\rm{W}}}_{{\rm{fill}}}$ in 2H-MX_2_ that it is crucially important to directly tune the Fermi level to enhance their voltages (Fig. 1C). Considering that Li introduced into the intercalation process is practically completely ionized via charge transfer (with the filled electron integrations in conduction bands, *e*^int^ > 0.880 e, Supplementary Table S2) and results in a rigid shift of the Fermi level (E_F_) (Fig. 1B), the energy acquired by the Li-2s electron transfer in systems is described as ${{\rm{W}}}_{{\rm{fill}}} = \mathop \smallint \nolimits_{{\rm E}_{\rm F}}^{{\rm E}^{\prime}} {\rm E}{{\rm \rho }}( \rm E ){\rm{d}}{\rm E}{\rm{\ }}$[20]. E is the energy referenced to the vacuum level, ${\rm{\rho }}( {\rm E} )$ is the density of state (DOS) of the system and ${\rm E}^{\prime}$ is derived from $\mathop \smallint \nolimits_{{\rm E}_{\rm F}}^{{\rm E}^{\prime}} {\rm{\rho }}( {\rm E} ){\rm{d}}{\rm E}{\rm{\ }} = {\rm{\ }}1$, which assumes that one Li-2s electron is transferred into the MX_2_ formula unit (LiMX_2_, Supplementary Table S2). It is also noted that MS_2_ and MSe_2_ have different slopes and intercepts in their individual linear relations, and the reasons are explained in Supplementary Section S2.

Besides, both the Fermi energy and W_fill_ decrease with the reduction in the M-element period, resulting in an average voltage limit of 1.321 V for LiCrS_2_ in the Group-VIB LiMX_2_ family (Fig. 1C). Considering that Group-VIB 2H-MX_2_ generally have band gaps of 0.7–1.2 eV and the ${\rm{e^{\prime}}}$ orbitals that dominate their VB exhibit fully occupied bonding characteristics (Supplementary Table S2), Li-2s electrons are expected to fill low-energy orbitals if holes are created beforehand, which may result in a higher voltage. A p-type alloying strategy for 2H-MX_2_ (X = S, Se) electrodes (with a higher-lying p-derived valence band and smaller ionization energies than oxides) is therefore proposed to achieve this goal. Here we emphasize that this strategy can be effectively used in a specific phase structure, i.e. a structure satisfying molecular-orbital transformation during charging/discharging, which is discussed in detail in Fig. 5. In this case, the Li-2s filling electron states can be transferred from CB in Group-VIB 2H-MX_2_ to the lower-energy-level states in the top region of VB in Group-VB 2H-M’X_2_, thereby increasing the electrode voltage (Fig. 1D). As a result, the average Li^+^-intercalation voltage increases significantly from 1.321 V in 2H-CrS_2_ to 2.435 V in 2H-VS_2_, which is attributed to the filling of Li-2s electrons into the low-energy ${\rm{e^{\prime}}}$ orbital in 2H-VS_2_.

### Identifying and quantitating the voltage-tuning/phase-stability competition

**Figure 2.**
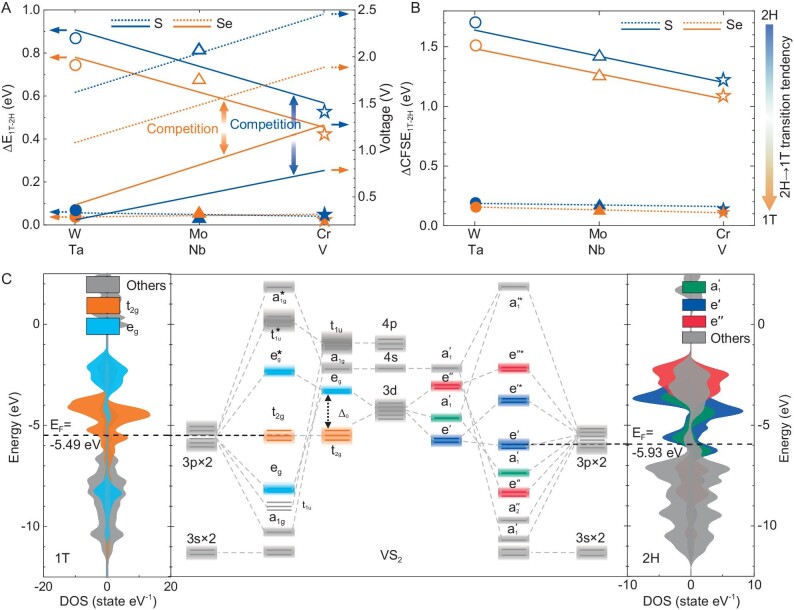
Theoretical foundation for quantitating voltage-tuning/phase-stability competition. The variation trend of (A) voltage (right axis), relative phase stability (ΔE_1T–2H_, left axis) and (B) crystal field stabilization energy difference between 2H- and 1T-phases (ΔCFSE_1T–2H_) of Group-VIB/VB MX_2_ with the change in the M-element period. The solid and dotted lines represent Group-VIB (W, Mo, Cr) and Group-VB (Ta, Nb, V) MX_2_ materials, respectively. The trends of the unsymbolized (voltage) and symbolized (ΔE_1T–2H_ and CFSE_1T–2H_) lines show crucial competitions between voltage tuning and phase stability. (C) Molecular-orbital energy diagrams that are automatically identified by our in-house-developed MOELD-LLF program [30, 49], as well as the corresponding DOS of 1T (left) and 2H (right) VS_2_.

Rather than the intended Li^+^-intercalation voltage enhancement, an undesired phase transition may occur in the system under p-type alloying, which may lead to Fermi-level reconstruction and thus voltage attenuation. Figure 2A shows a basic competition between voltage tuning and phase stability, i.e. the reduction in the M-element period leads to an increase in the system voltage, but is also accompanied by a phase transition of the system from 2H to 1T. In fact, VS_2_ undergoes a phase transition from 2H to 1T above ∼270 K [21,22], causing the attenuation of the average voltage of Li*_m_*VS_2_ (0 ≤ *m* ≤ 1) from 2.435 V in 2H to 2.067 V in 1T (Supplementary Fig. S5). Same phenomenon also occurs in the TiS_2_ electrode, which is already in 1T-phase at 0 K with a Li*_m_*TiS_2_ average voltage of ∼2.1 V [7]. It inspires us to analyse the essential reasons for the 2H–1T phase transition based on ligand-field theory. We postulate that the Fermi-level position of the electrode is mainly determined by two aspects: (i) the splitting strength of the M-d orbitals [23], Dq$= \frac{{{Z}_L{e}^2\langle {r}^{4}\rangle}}{{6{R}^5}} $, where *Z_L_* is the charge on the ligand, *R* is the average M-ligand distance and $\langle {r}^4\rangle $ is the mean nucleus–electron radial distance exponentiated to the fourth power, which is usually replaced by the ionic radius (${r}_i$) (Supplementary Section S3.1); (ii) the M-d splitting coefficient determining the Fermi level, *n* (Supplementary Table S3). Accordingly, we propose a descriptor, $\Delta _{{\rm{\alpha }} - {\rm{\beta }}}^{{\rm{CFSS}}}$, to compare the Fermi levels of the specified α and β phases:

(1) \begin{equation*}\Delta _{{\rm{\alpha }} - {\rm{\beta }}}^{{\rm{CFSS}}} = {n}_{\rm{\alpha }}\frac{{{Z}_L{e}^2{r}_i^4}}{{6R_{\rm{\alpha }}^5}} - {n}_{\rm{\beta }}\frac{{{Z}_L{e}^2{r}_i^4}}{{6R_{\rm{\beta }}^5}}. \end{equation*}

It is suggested that in Group-VIB MX_2_ electrodes, due to the saturation of the lower-energy-level e′ orbitals of their 2H phases, Li-2s electrons are forced to jump cross the band gap to fill into the higher-energy-level ${\rm{e}}{{\rm{^{\prime}}}}^{\rm{*}}$ orbitals during lithiation (as partial density of state characteristics of CrS_2_ shown in Fig. 1D). This results in the lower voltages of Group-VIB MX_2_ with 2H phase structures than that with 1T phase structures, where Li-2s were filled into ${{\rm{t}}}_{2{\rm{g}}}$ orbitals (Fig. 2A and Supplementary Fig. S6A). This can be also quantitatively verified by the proposed crystal field-splitting strengths (CFSS) descriptor. As can be seen from Supplementary Fig. S6B, $\Delta _{1{\rm{T}} - 2{\rm{H}}}^{{\rm{CFSS}}}$ of all Group-VIB MX_2_ electrodes are all negative, resulting in the average Li^+^-intercalation voltages of 2H phases generally being 0.45–0.90 V lower than those of 1T phases. Even so, special attention should be paid here that due to the high energy-level limit of the t_2 g_ ligand-field splitting in 1T phases, their average voltage cannot exceed 1.823 V in 1T-CrS_2_. On the contrary, the voltages of Group-VB MX_2_ with 2H phase structures are significantly higher than that with 1T phase structures (0.08 V ≤ $\Delta {{\rm{V}}}_{1{\rm{T}} - 2{\rm{H}}}$ ≤ 0.40 V, Supplementary Fig. S6A). This is essentially because the CFSS of 2H phases are larger than those of the 1T phases, resulting in their more negative CFSS values and correspondingly deeper Fermi levels (Supplementary Fig. S6B, $\Delta _{1{\rm{T}} - 2{\rm{H}}}^{{\rm{CFSS}}}$ are positive for all Group-VB MX_2_). For example, the Fermi-level position difference between 1T and 2H VS_2_ is $\Delta _{1{\rm{T}} - 2{\rm{H}}}^{{\rm{CFSS}}} = - 4{\rm{\ }} \times \frac{{{Z}_L{e}^2{r}_i^4}}{{6R_{1{\rm{T}}}^5}} - ( { - 5.84} ) \times \frac{{{Z}_L{e}^2{r}_i^4}}{{6R_{2{\rm{H}}}^5}} = 0.068{\rm{\ eV}}$, indicating the relatively higher Fermi-level energy of 1T-VS_2_ (Fig. 2C). This is the fundamental reason for the undesired voltage attenuation caused by the 2H–1T phase transition in MX_2_ materials. Moreover, the instability of the above high-voltage 2H-MX_2_ is closely related to the crystal field splitting of the M-center in the system, which contributes to the crystal field stabilization energy (${\rm{CFSE}} = ( {( {{n}_1{N}_x + {n}_2{N}_y + \cdot \cdot \cdot {\rm{\ }} \cdot \cdot \, \cdot } ){\rm{Dq}} + m{\rm{P}} + \frac{{2( {{\rm{\delta }} + {\rm{\sigma }}} )}}{3}} )$) and the electronic configurational entropy, S_CFS_: ${{\rm{G}}}^{{\rm{CFS}}} = - {\rm{CFSE}} - {\rm{T\ }} \times {{\rm{S}}}_{{\rm{CFS}}}$ [24]. Here the entropy contribution in ground state (0 K) is negligible. The term $\frac{{2( {{\rm{\delta }} + {\rm{\sigma }}} )}}{3}$ is the extra stabilization enthalpy gained by M-ions as a result of the MX_6_ distortion, which can be ignored in MX_2_ with the same structure. Besides, the *m*-factor in front of pairing energy (P) depends on the number of forced pairing electrons (see Supplementary Table S4 and 9 for different values of ligand-field systems, respectively), which is not equal to 0 for d*^q^* (*q* ≥ 4) systems, such as the strong field case for the d^4^ complex shown in Supplementary Fig. S7. We also emphasize that the electronic configurations of the M-ions in Group-VB/VIB MX_2_ systems are ${{\rm{d}}}^1/{{\rm{d}}}^2$, no paired energy (P) is produced. In this case, the relative energy stability of specific $\alpha$ and $\beta$ phases (${\rm{\Delta G}}_{{\rm{\alpha }} - {\rm{\beta }}}^{{\rm{CFS}}}$) can be approximated by using an improved descriptor (${\rm{\Delta CFS}}{{\rm{E}}}_{{\rm{\alpha }} - {\rm{\beta }}}$, Supplementary Section S3.2):

(2) \begin{eqnarray*} &&{{\rm{\Delta CFS}}{{\rm{E}}}_{{\rm{\alpha }} - {\rm{\beta }}}}\\ && = {\left( {{n}_1{N}_x + {n}_2{N}_y + \cdot \cdot \cdot {\rm{\ }} \cdot \cdot \, \cdot } \right)}_{\rm{\alpha }} \times {\left({{\rm{g}}}^{\prime} \times \frac{{{Z}_L{e}^2{r}_i^4}}{{6{R}^5}}\right)}_{\rm{\alpha }}\\ && - \big( {{n}_1{N}_x + {n}_2{N}_y} + { \cdot \cdot \cdot {\rm{\ }} \cdot \cdot \cdot \cdot } \big)_{\rm{\beta }} \times {\left({{\rm{g}}}^{\prime} \times \frac{{{Z}_L{e}^2{r}_i^4}}{{6{R}^5}}\right)}_{\rm{\beta }},\\ \end{eqnarray*}

where ${n}_i$ (*i* = 1, 2, …) represent the symmetry coefficients of different orbitals (Supplementary Table S3), ${N}_m$ (*m* = *x, y*, …) are the electron numbers in the above orbitals and ${\rm{g^{\prime}}}$ represents a spectrochemical series of central ions for the same ligand (Supplementary Section S3.2). The larger the ${\rm{\Delta CFS}}{{\rm{E}}}_{{\rm{\alpha }} - {\rm{\beta }}}$, the lower the ${\rm{\alpha }} \to {\rm{\beta }}$ phase-transition tendency. It is seen from Fig. 2B that ${\rm{CFS}}{{\rm{E}}}_{1{\rm{T}} - 2{\rm{H}}}$ gradually decreases as the M-period number decreases, suggesting that the MX_2_ systems have increased 2H–1T phase-transition tendencies from W to Cr. Besides, the crystal field stabilization energy differences between 2H- and 1T-phases (${\rm{\Delta CFS}}{{\rm{E}}}_{1{\rm{T}} - 2{\rm{H}}}$) of Group-VB MX_2_ electrodes are generally small (0.10–0.20 eV, Fig. 2B), even one order of magnitude smaller than those of Group-VIB MX_2_ electrodes (1.20–1.80 eV). These are consistent with the total energy differences between 2H and 1T structures calculated by using first principles ($\Delta {{\rm{E}}}_{1{\rm{T}} - 2{\rm{H}}}$, Fig. 2A). Thus, we emphasize that the two descriptors, $\Delta _{1{\rm{T}} - 2{\rm{H}}}^{{\rm{CFSS}}}$ and ${\rm{\Delta CFS}}{{\rm{E}}}_{1{\rm{T}} - 2{\rm{H}}}$, provide a theoretical foundation to quantitatively balance the voltage-tuning/phase-stability competition under p-type alloying.

### Ions-intercalation behaviors of 2H-V_1.__75_Cr_0__.25_S_4_

**Figure 3.**
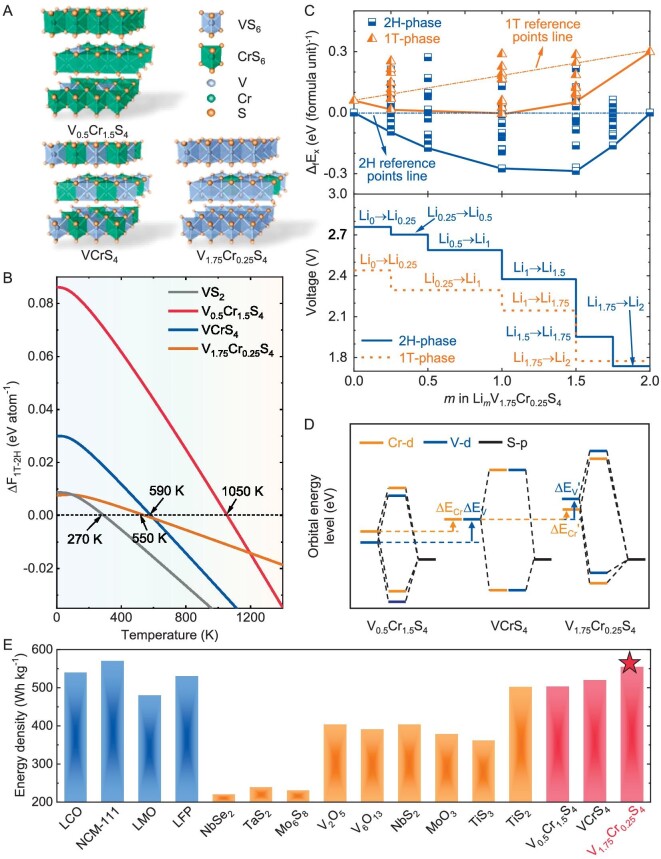
Theoretical Li^+^-intercalation behaviors of 2H-V*_x_*Cr_2−_*_x_*S_4_. (A) Crystal structures of 2H-V*_x_*Cr_2−_*_x_*S_4_ (*x* = 0.5, 1, 1.75). (B) Free energy difference between 1T- and 2H-V*_x_*Cr_2−_*_x_*S_4_ (ΔF_1T–2H_). The 2H→1T phase-transition temperatures are predicted to be 270, 550, 590 and 1050 K for VS_2_, V_1.75_Cr_0.25_S_4_, VCrS_4_ and V_0.5_Cr_1.5_S_4_, respectively. (C) Formation energies (Δ_f_E_x_) and voltages of 1T- and 2H-V_1.75_Cr_0.25_S_4_ are shown as a function of Li concentration (Li*_m_*V_1.75_Cr_0.25_S_4_). Yellow and blue solid lines indicate the constructed convex hull of the 1T-phase and 2H-phase, respectively. For each Li concentration point on the convex hull, we only list the lowest 10 configurational energies. (D) The V-d (blue lines) and Cr-d (yellow lines) relative energy-level changes in V_0.5_Cr_1.5_S_4_/VCrS_4_ and VCrS_4_/V_1.75_Cr_0.25_S_4_ are represented by ΔE_V_ (ΔE_V_') and ΔE_Cr_ (ΔE_Cr_'), respectively. (E) The energy densities (at the electrode level) for a series of fully or partially commercialized intercalation-type Li-containing cathodes (blue), common Li-free cathodes (yellow) and 2H-V*_x_*Cr_2−_*_x_*S_4_ (red), calculated based on their specific capacities (mAh g^−1^) and average discharge voltages (V vs. Li/Li^+^). The capacities are either for fully lithiated hosts (blue) or lithium-free (yellow and red) hosts. LCO, LiCoO_2_; NCM-111, Li[Ni_0.333_Co_0.333_Mn_0.333_]O_2_; LMO, LiMn_2_O_4_; LFP, LiFePO_4_.

The above results show that CrS_2_ is the material with a stable 2H structure at room temperature and lowest E_F_ (−0.534 eV, Supplementary Table S2) in Group-VIB/VB 2H-MX_2_ (Fig. 2B). Being adjacent to Cr in the periodic table, V is an ideal dopant for creating holes in 2H-CrS_2_ according to our electronic p-type alloying strategy. In this way, the 2H phase stability of the MX_2_ electrode is guaranteed and the high voltage could be achieved competitively. Using the particle swarm optimization technique implemented in the CALYPSO code [25], we identify the stable structures in the V*_x_*Cr_2−_*_x_*S_4_ (0 ≤ *x* ≤ 2) system. The effectiveness of CALYPSO in finding layered materials has been validated by successful reproduction of known materials [26]. Following the procedure described in Supplementary Section S4, we reproduce the known 2H-CrS_2_ structure and identify a new stable 2H-phase V–Cr–S family: V_0.5_Cr_1.5_S_4_ (*Cmcm*), VCrS_4_ (*Pmmn*) and V_1.75_Cr_0.25_S_4_ (*Amm2*) (Fig. 3A).

The thermodynamic and kinetic stabilities of the identified phases are further verified by calculating the grand potential phase diagram for the ground states (Supplementary Table S6), as well as by conducting phonon calculations and *ab initio* molecular dynamics (AIMD) simulations. To construct the phase diagram, we extract all structural prototypes and energies in the V–Cr–S ternary system from the Inorganic Crystal Structure Database (ICSD) [27]. Supplementary Fig. S8 shows that all 2H-phase V_0.5_Cr_1.5_S_4_, VCrS_4_ and V_1.75_Cr_0.25_S_4_ are located on the formation energy convex hull, indicating their thermodynamic stability. Also, no imaginary frequencies are found in the phonon-dispersion curves, indicating their dynamic stabilities (Supplementary Section S5 and Supplementary Fig. S9). In addition, all structures are well preserved during AIMD simulations at 400 K, suggesting that they will remain stable at the cell-operating temperatures (Supplementary Fig. S10).

The phonon spectra and vibrational entropies of V_0.5_Cr_1.5_S_4_, VCrS_4_ and V_1.75_Cr_0.25_S_4_ are calculated using quasi-harmonic approximation to further explore the effect of temperature on the relative phase stabilities of 2H- vs. 1T-phases (Supplementary Fig. S9). The free energies of 1T-V*_x_*Cr_2−_*_x_*S_4_ decrease faster than those of 2H-V*_x_*Cr_2−_*_x_*S_4_ as the temperature increases due to the vibration entropies of all 1T-phases obviously being larger than those of 2H-phases at finite temperature (Supplementary Fig. S11). Among them, V_0.5_Cr_1.5_S_4_ exhibits the highest 2H→1T phase-transition temperature (1050 K, Fig. 3B), which well explains that it has been experimentally discovered and used as a cathode material for lithium batteries [28]. However, it should be emphasized that the addition of V creates more holes, which is beneficial to enhance the voltage and energy density, but also leads to a decrease in the phase-transition temperature (Fig. 3B). Therefore, by tuning 2H-V*_x_*Cr_2−_*_x_*S_4_ electrodes using a p-type alloying strategy, we identify the newly designed 2H-V_1.75_Cr_0.25_S_4_ as an optimal candidate in terms of the balance between voltage-tuning and phase-stability competition. However, the synthesis challenge of 2H-V_1.75_Cr_0.25_S_4_ due to its narrow preparation temperature range (lower than its phase-transition temperature, 550 K) will be discussed in the following part.

As we further investigate the electronic structure of 2H-V*_x_*Cr_2−_*_x_*S_4_ (*x* = 0.5, 1, 1.75), it achieves the goal of creating unoccupied states close to VB, and eventually causes the Li-2s to fill the much lower ${\rm{e^{\prime}}}$ bonding orbitals (Fig. 1D). As a result, W_fill_ of 2H-phase V_0.5_Cr_1.5_S_4_, VCrS_4_ and V_1.75_Cr_0.25_S_4_ shift to −2.88, −2.92 and −3.03 eV, respectively, and are much lower than those of Group-VIB 2H-MS_2_ (Supplementary Fig. S12). Moreover, the Fermi level passes through VB of 2H-V*_x_*Cr_2−_*_x_*S_4_, proving the success of our p-type alloying strategy in creating holes in valence bands. Furthermore, after a rigorous search on 2H/1T-Li*_m_*V*_x_*Cr_2−_*_x_*S_4_ (0 ≤ *m* ≤ 2) with unit cells of ≤32 Li sites for different intercalation stages, the ground-state hulls for both 2H- and 1T-phases are established. Here, if we take 2H/1T-Li*_m_*V_1.75_Cr_0.25_S_4_ (Fig. 3C) and 2H/1T-Li*_m_*V_0.5_Cr_1.5_S_4_ (Supplementary Fig. S13) as examples, the formation energies (${\Delta }_{\rm{f}}{{\rm{E}}}_{\rm{x}}$) of 1T-phases are found to be higher than those of 2H-phases across the entire composition ranges in both cases, indicating their instabilities during lithiations. As a result, the initial Li^+^-intercalation voltages of 2H-phase Li*_m_*V_0.5_Cr_1.5_S_4_, Li*_m_*VCrS_4_ and Li*_m_*V_1.75_Cr_0.25_S_4_ are 2.623, 2.702 and 2.767 V, respectively, confirming that tunings of Fermi levels indeed dramatically enhance the voltages of 2H-phases as compared to those of 1T-phases (Fig. 3C and Supplementary Fig. S13) and pristine Group-VIB 2H-MS_2_ (Supplementary Fig. S12 and Supplementary Table S7). Notably, the calculated 2H-V_0.5_Cr_1.5_S_4_ open-circuit voltage is also highly consistent with the earlier experimental result, where the energy density of the previously synthesized V_0.5_Cr_1.5_S_4_ cathode [28] is theoretically verified to be 502.6 Wh kg^−1^ at the electrode level (Supplementary Table S7). Most importantly, the theoretical energy density of V_1.75_Cr_0.25_S_4_ reaches 554.3 Wh kg^−1^, representing a record value in the existing intercalation-type Li-free cathode field and comparable to that of traditional Li-containing cathodes [29] (Fig. 3E). Here the specific energy density (energy per unit mass, Wh kg^−1^) is calculated by integrating the operating voltage (E, V) over the specific capacity (Q, charge amount per unit mass, mAh g^−1^) in Fig. 3E: specific energy density $= \smallint {\rm{EdQ}}$.

We next uncover the characteristics of electrochemical active centers that dominate the capacities of 2H-V*_x_*Cr_2−_*_x_*S_4_ (*x* = 0.5, 1, 1.75) cathodes during lithiation. The electronic structures of 2H-V*_x_*Cr_2−_*_x_*S_4_ are highly dependent on the d-p hybridization of the transition metal (Cr/V) and ligands (S), which are strictly governed by their local ligand fields (Supplementary Fig. S14A). Because the orbital energies of V/Cr-3d and S-3p are close, U/2 (orbital hybridization degree) ≈ Γ (the difference between atomic-orbital energy levels) in 2H-V*_x_*Cr_2−_*_x_*S_4_ (Supplementary Fig. S14B) [30]. Thus, there are strong covalent characteristics between them, which are beneficial for increasing specific capacity and redox reversibility. In this case, both V/Cr-3d and S-3p locate at the Fermi level; electrons introduced in the lithiation process should be accommodated in Cr/V-3d and S-3p hybridization states. Here the relative positions of ${\rm{e^{\prime}}}$ orbitals in 2H-V*_x_*Cr_2−_*_x_*S_4_ (*x* = 0.5, 1, 1.75) that determine the valence-state changes are predicted by a ligand-field-related descriptor ($- 5.84{\rm{\ }} \times \frac{{{Z}_L{e}^2{r}_i^4}}{{6{R}^5}}$, Supplementary Table S3). A larger splitting energy ($\frac{{{Z}_L{e}^2{r}_i^4}}{{6{R}^5}}$) would result in a lower energy of the Cr (or V) ${\rm{e^{\prime}}}\ $levels (Supplementary Fig. S14C). It is found that when moving from 2H-V_0.5_Cr_1.5_S_4_ to 2H-V_1.75_Cr_0.25_S_4_, the rise in V-${\rm{e^{\prime}}}$ (denoted as $\Delta {{\rm{E}}}_{\rm{V}}$ or $\Delta {{\rm{E}}}_{\rm{V}}{\rm{^{\prime}}}$) is faster than that of Cr-${\rm{e^{\prime}}}$ ($\Delta {{\rm{E}}}_{{\rm{Cr}}}$ or $\Delta {{\rm{E}}}_{{\rm{Cr}}}{\rm{^{\prime}}}$, Supplementary Fig. S14D), resulting in the dominated participation of V-3d/S-3p during lithiation of the 2H-V_1.75_Cr_0.25_S_4_ cathode (Fig. 3D and Supplementary Fig. S15).

To achieve good cycling performance, volume changes of within 20% for electrodes are suggested [31]. The calculated expansion coefficient for 2H-V_1.75_Cr_0.25_S_4_ after lithiation is 10.91% (Supplementary Section S6 and Supplementary Fig. S16), which is much lower than that of conversion reaction electrodes (e.g. volume expansion of Si-anode reaching 300% [32]), demonstrating its advantages to bear the strain during charging/discharging. According to the empirical Pugh formula [33], the lower G/B (∼0.59) and higher Poisson ratios (∼0.25) indicate that 2H-V_1.75_Cr_0.25_S_4_ is less brittle (Supplementary Fig. S16 and Supplementary Table S8), confirming its high structural stability and reversibility. The Li-ion diffusion behavior in 2H-V_1.75_Cr_0.25_S_4_ under fully charged and discharged states is also investigated (Supplementary Fig. S17). The calculated Li^+^ diffusion barriers in Li_2__–_*_m_*V_1.75_Cr_0.25_S_4_ (0 ≤ *m* ≤ 2) and V_1.75_Cr_0.25_S_4_ are 0.456 and 0.452 eV, respectively, close to those in common cathodes (<0.6 eV) that have been widely used in battery applications [34].

### Verification of the voltage advantage of 2H-V_1.75_Cr_0.25_S_4_

As proof of the p-type alloying strategy, V_1.75_Cr_0.25_S_4_ materials with various phase characteristics were prepared by using a liquid-phase chemical method to verify the voltage advantage of the 2H phase. Previous reports on vanadium-based sulfides suggest that common synthesis temperatures (>430 K) invariably generate 1T-phase products [35]. Accordingly, two synthesis temperatures were respectively set at 450 and 400 K for obtaining distinct phase contents, when the corresponding products (denoted VCS-450 and VCS-400) were 1T and 1T + 2H-mixed phases, respectively. In fact, a series of hydrothermal temperatures (from 380 to 420 K) have been tried to prepare V_1.75_Cr_0.25_S_4_, confirming that 400 K is the temperature limit for successful preparation (Supplementary Fig. S18). On one hand, the existence of the 2H-phase in VCS-400 is attributed to its largely lower synthetic temperature than those of all the previous works on the liquid-phase synthesis of vanadium-based sulfides (Supplementary Table S9), which delays the product-phase transition from 2H to 1T to some extent. On the other hand, the 2H-phase only exists in the form of a mixed phase rather than a pure phase because the synthetic temperature cannot be further lowered, for guaranteeing adequate energy fluctuation in the nucleation process of sulfide [36]. The reason for the lower actual synthesis temperature for experimentally observing 1T-V_1.75_Cr_0.25_S_4_ than our theoretically predicted 2H–1T transition temperature (550 K, Fig. 3B) comes from the high-pressure environment in liquid-phase synthesis. In fact, limited by the available synthesis methods of metal sulfides related with high temperature and/or high pressure, bulk pure 2H-phase vanadium-based sulfide has not been reported until now [37,38].

**Figure 4.**
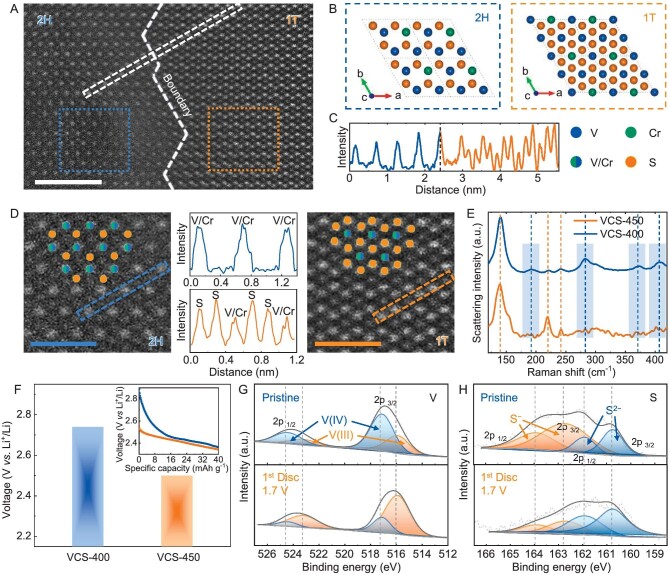
Verification of Li^+^-intercalation voltage advantage of 2H-V_1.75_Cr_0.25_S_4_. (A) STEM-HAADF image of 2H/1T-phases coexistence region for VCS-400. Scale bar: 2 nm. (B) Crystal structures of 2H and 1T-phase V_1.75_Cr_0.25_S_4_ viewed from the [001] crystallographic direction. (C) The line profile crossing the 2H/1T-phase boundary. (D) Selected area STEM images of 2H and 1T regions and corresponding line profiles. Scale bars: 1 nm. (E) Raman spectra of VCS-400 and VCS-450. (F) The initial discharge voltage of cells based on VCS-400 and VCS-450 cathodes (inset: discharge curves at initial stage). (G) V 2p and (H) S 2p state X-ray photoelectron spectroscopy (XPS) spectra of VCS-400 before and after discharging to 1.7 V.

Both VCS-400 and VCS-450 samples demonstrated a 3D hierarchical structure accompanied with a uniform distribution of V, Cr and S elements, while the Cr/V ratios matched with the theoretical values to indicate a successful doping of Cr into a V-based matrix (Supplementary Section S7A and B, and Supplementary Fig. S19). High-angle annular dark field-scanning transmission electron microscopy (HAADF-STEM) was employed to visualize the atomic arrangement. As expected, both 2H- and 1T-phase regions coexisted with clear boundaries in VCS-400 (Fig. 4A), being consistent with corresponding crystal structures viewed from the [001] crystallographic direction (Fig. 4B). Meanwhile the line profile crossing two regions gave lattice spacings in agreement with the (001) planes of 2H- and 1T-V_1.75_Cr_0.25_S_4_ simultaneously (Fig. 4C). This clearly verified the existence of 2H-phase in a 2H/1T mixed-phase system, which is further confirmed by selected-area STEM and high-resolution transmission electron microscopy (HRTEM) (Fig. 4D and Supplementary Fig. S20). Moreover, the differential scanning calorimetry (DSC) curve of VCS-400 gave an exothermic peak at >500 K quite close to the theoretical 2H–1T phase-transition point, as compared with VCS-450 without obvious phase-transition behavior which indicated a pure 1T-phase (Fig. 3B and Supplementary Fig. S21). The characteristic bands (192, 283, 371 and 407 cm^−1^) emerging in the Raman spectrum of VCS-400 were assigned to the vibration modes of 2H-V_1.75_Cr_0.25_S_4_ based on our calculation results (Fig. 4E and Supplementary Fig. S22). The XRD peaks of both samples matched well with the calculated pattern of the $P\bar{3}m1$ space group of the 1T-phase. The relatively low content of 2H-phase in VCS-400 resulted in the absence of obvious characteristic XRD peaks (Supplementary Section S7C and Supplementary Fig. S23), but the combination of the above techniques sensitive at the nanoscale verified the existence of 2H-V_1.75_Cr_0.25_S_4_ in VCS-400 prepared at a lower reaction temperature, albeit in the form of a 1T/2H mixed phase.

We assembled Li-metal batteries to verify the voltage enhancement of p-type alloying of 2H-V_1.75_Cr_0.25_S_4_. It was found that the VCS-400 cell possessed a higher voltage over VCS-450, 1T-VS_2_ and commercial TiS_2_ during the initial discharge stage, i.e. its initial voltage (discharge to 1% capacity) reached 2.74 V, which is quite close to the theoretical value of 2H-V_1.75_Cr_0.25_S_4_ (2.767 V) and substantially higher than those of VCS-450 (2.50 V) and 1T-VS_2_ (2.52 V) (Fig. 4F and 9). The galvanostatic intermittent titration technique tests also gave similar conclusions (Supplementary Fig. S25). Although the discharge curves of the V-based cathode cells are mainly dominated by 1T-phase, the higher initial Li^+^-intercalation voltage of VCS-400 benefits from existing 2H-phase V_1.75_Cr_0.25_S_4_, which possesses voltage advantages over 1T-V_1.75_Cr_0.25_S_4_ and 1T-VS_2_. The *in situ* XRD patterns of VCS-400 indicate that no obvious conversion reaction occurs at >1.7 V, confirming the intercalation-type Li-storage mechanism of V_1.75_Cr_0.25_S_4_ accompanied by high structural stability (Supplementary Fig. S26). In addition, the voltage advantage of 2H-V_1.75_Cr_0.25_S_4_ is also verified by solid-state batteries (Supplementary Fig. S27).

When galvanostatic discharge–charge cycled at 0.1 C within a voltage range of 1.7–2.8 V, both the VCS-400 and VCS-450 cathodes delivered high initial discharge capacities (∼230 mAh g^−1^) close to theoretical values and high coulombic efficiencies at room temperature (Supplementary Section S7D and Supplementary Fig. S28). The high-temperature tests at 55°C also demonstrated good cycling stability (Supplementary Fig. S29). Meanwhile, their low volume-expansion degrees (4.1% for VCS-400 and 4.7% for VCS-450) after initial lithiation evidence superior structural stability attributed to its intercalation-type lithium-storage mechanism with an intrinsic low expansion coefficient (Supplementary Section S7E and Supplementary Fig. S30) [39]. In addition, they possess similar rate capability because of the same theoretical specific capacities (Supplementary Fig. S31). The above electrochemical performance demonstrates that the customized 2H-V_1.75_Cr_0.25_S_4_ enables it to reach the optimal balance between voltage tuning and phase stability, which renders the application value of MX cathodes based on the p-type alloying strategy. In addition, the valence-state changes of VCS-400 during the Li^+^-intercalation stage indicated the redox center roles for both the V cations and S anions, in good agreement with our first-principles calculations (Supplementary Section S7F, and Fig. 4G and H).

## DISCUSSION

As mentioned in the introduction, the key bottleneck currently limiting all-solid-state Li-metal battery performance is the resistance at the cathode/electrolyte interfaces. The traditional oxide cathode/sulfide electrolyte interfaces have a universal difficulty in Li^+^ conduction [40]. Thus, we systematically calculate and compare the space charge layer effect at interfaces between fully discharged 2H-Li_2_V_1.75_Cr_0.25_S_4_ cathode/Li_3_PS_4_ electrolyte (LVCS/LPS, Supplementary Fig. S32A) and traditional LiCoO_2_ cathode/Li_3_PS_4_ electrolyte (LCO/LPS, Supplementary Fig. S32B) systems. Details of the reasons for choosing the LPS electrolyte and the above interface modeling are described in Supplementary Section S8. The LCO/LPS crystal structure is largely distorted (Supplementary Fig. S32B). The LPS has been adsorbed on the LCO side with a large Li-vacancy formation energy (E_v_ = 3.27 eV, Supplementary Table S10), indicating that Li^+^ adsorption on the LCO cathode is more favorable than that on the underground region of the LPS electrolyte. This results in a high interfacial Li^+^ concentration (*C*_Li_), and therefore a large interfacial resistance at both the equilibrium (Supplementary Fig. S32D) and initial charging stages (Supplementary Fig. S32F). By contrast, E_v_ of LVCS/LPS changes gently with a downward trend in the interface region, thus providing smooth Li^+^ migration paths free from possible bottlenecks and reducing the interfacial resistance at both the equilibrium (Supplementary Fig. S32C) and initial charging stages (Supplementary Fig. S32E). We suggest that the low interfacial resistance found at LVCS/LPS is essentially attributed to their physico-chemical compatibility. The S atoms at the interface exhibit an S-3p atomic-orbital nature due to the serious lack of coordination, ultimately leading to the smooth matching of the LVCS/LPS interface (Supplementary Fig. S32A). The current discussion on the interfacial structures can be generally applied to the other sulfide cathodes, thus opening up a new direction to address the challenges posed by the interface between traditional oxide cathodes and electrolytes in all-solid-state batteries.

**Figure 5.**
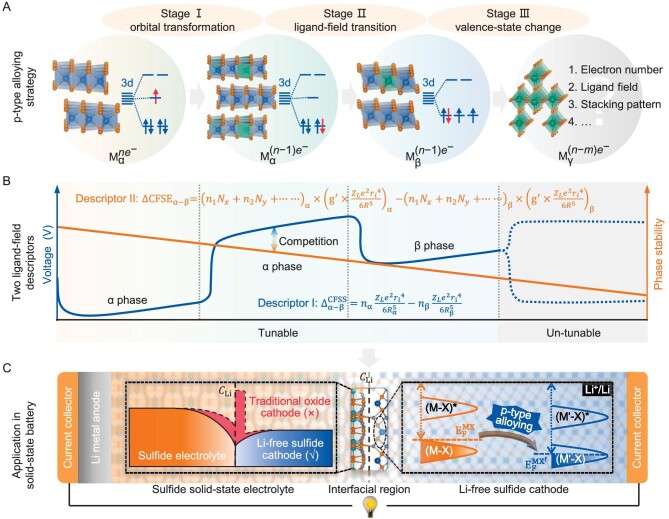
Three phase/valence evolution stages involved in p-type alloying strategy. (A and B) The competition between voltage tuning (blue lines) and phase stability (yellow lines) of MX in three phase/valence evolution stages (I, II and III) under p-type alloying are quantitated by two improved ligand-field descriptors ($\Delta _{{\rm{\alpha }} - {\rm{\beta }}}^{{\rm{CFSS}}}{\rm{\ and\ }}\Delta {\rm{CFS}}{{\rm{E}}}_{{\rm{\alpha }} - {\rm{\beta }}}$). *n* and *m* represent the M valence electrons; α, β and $\gamma;$ represent different MX phases. (C) The p-type alloying strategy has general implications for the design of high-voltage Li-free M’X cathodes to pair with an Li-metal anode to improve the energy density of all-solid-state Li-metal batteries (the inset on the cathode side). Simultaneously, as shown in the inset on the solid-state electrolyte side, these newly designed sulfide cathodes have great potential to have better cathode/electrolyte physico-chemical compatibility (a uniform Li^+^ distribution, *C*_Li_, at the interface, colored solid area) than traditional Li-containing oxide cathodes (red dotted area) for all-solid-state Li-metal batteries.

Furthermore, we suggest that the voltage and phase-stability evolution of electrodes involved in the proposed p-type alloying strategy can be broadly summarized into three stages (Fig. 5A) based on two quantitative descriptors ($\Delta _{{\rm{\alpha }} - {\rm{\beta }}}^{{\rm{CFSS}}}$ and ${\rm{CFS}}{{\rm{E}}}_{{\rm{\alpha }} - {\rm{\beta }}}$). In Stage I, the electrodes are tuned by the p-alloying strategy, which creates unoccupied states close to VB and shifts down the Fermi levels of systems. This enables the transformation of Li^+^ electrons into a lower-lying orbital (Stage I, Fig. 5A), leading to a higher voltage. In this case, the system may undergo an undesired phase transition (Stage II), thus leading to a reconstruction of the Fermi level and voltage attenuation (voltage-tuning/phase-stability competition, Fig. 5B). This well explains the experimental observation of the 2H–1T phase transition at ∼270 K in VS_2_, resulting in the average Li^+^-intercalation voltage decaying from 2.435 V (2H-VS_2_) to 2.067 V (1T-VS_2_, Supplementary Fig. S5). Besides, the p-type alloying may also lead to the change of the transition metal to a higher valence state (Stage III), which is highly dependent on the local ligand fields and stacking patterns of electrodes (Fig. 5B). In fact, many novel electrodes reported adherence to this tuning paradigm. Recent works have shown that high-rate capabilities can be achieved in complex Wadsley–Roth phases Nb_12_WO_33_, Nb_14_W_3_O_44_ and Nb_16_W_5_O_55_ electrodes [41]. In this case, these compounds are composed of complex corner-sharing octahedra blocks and high valence states of Nb (+5) and W (+6) to stabilize structures. From this, the proposed p-type alloying strategy has customized implications for designing Li-free cathodes for all-solid-state Li-metal batteries based on a Fermi-level-dominated energy-storage mechanism (Fig. 5C).

## CONCLUSIONS

In this work, we explore the crucial voltage-tuning vs. phase-stability competition that has previously been overlooked in cathode systems. We propose a p-type alloying strategy involving three interconnected stages: molecular-orbital transformation, ligand-field transition and transition-metal valence-state change. Each regime is quantitatively characterized by the two improved ligand-field descriptors, i.e. $\Delta _{{\rm{\alpha }} - {\rm{\beta }}}^{{\rm{CFSS}}}$ and ${\rm{CFS}}{{\rm{E}}}_{{\rm{\alpha }} - {\rm{\beta }}}$, which allow adjustment of the voltage vs. phase-stability balance to achieve an ideal voltage. This p-type alloying strategy is independent of specific electrode materials. Following this, an intercalation-type 2H-V_1.75_Cr_0.25_S_4_ cathode with an initial Li^+^-intercalation voltage reaching 2.767 V and a record-breaking theoretical energy density of 554.3 Wh kg^−1^ is successfully designed. It is also emphasized that the 2H-V_1.75_Cr_0.25_S_4_ cathode and Li_3_PS_4_ solid-state electrolyte interface provides a smooth Li^+^ migration path, thus exhibiting a lower interfacial resistance than traditional oxide electrodes. Subsequent experimental results confirm its superior voltage and energy density. This work presents a customized strategy in designing sulfide cathodes for all-solid-state Li-metal batteries through electronic band-structure engineering, which helps to overcome the high reliance of current commercial cathodes on Co/Ni with high cost, scarcity and a centralized/volatile supply chain.

## METHODS

### General information

Additional details of materials and diversity of employed characterization techniques are presented in the Supporting information.

### Density functional theory calculations

All the calculations are performed based on the density functional theory (DFT) as implemented in the Vienna ab initio simulation package [42]. The core electrons are treated via the projector augmented wave method; an electron exchange-correlation effect is described using the generalized gradient approximation of the Perdew–Burke–Ernzerhof functional [43]. The plane-wave cut-off is 550 eV. The first Brillouin zone is sampled using a Monkhorst–Pack special k-point mesh; the density of the k-mesh ensures that the interval of the grid is <0.03 Å^−1^. The vdW-D2 method has been used to better estimate the layer-spacing values [44]. Spin polarization was considered in all the computations. The optimal Li-migration pathways and the migration energy barriers were obtained by using the nudged elastic-band method [45]. The phonon frequencies were calculated using the PHONOPY package [46]. AIMD simulations were carried out using the canonical (NVT) ensemble, which is controlled with a Nosé–Hoover thermostat [47]. After V*_x_*Cr_2−_*_x_*S_4_ (0 ≤ *x* ≤ 2) systems reached their equilibrium states, the temperature was kept at ∼400 K for 8 ps (4000 MD steps). The Gibbs free energies of V*_x_*Cr_2−_*_x_*S_4_ were calculated by using the temperature-dependent effective potential technique [48]. Besides, we realized the identification of the atomic-orbital splitting according to the local coordination environment of V–Cr–S cathodes, and further determined the molecule orbitals of systems through our in-house-developed program: Identifying Local Coordination Fields to Construct Crystal Molecular Orbital Energy Levels (MOELD-LLF). The program is open-source (access address: https://gitlab.com/shuhebing/MOELD-LLF) [30,49]. The calculation workflow was managed by the high-throughput computational platform for battery materials [50].

### Materials and synthesis

V_1.75_Cr_0.25_S_4_ samples (VCS-450 and VCS-400) were prepared by using the hydrothermal process using a certain amount of Na_3_VO_4_, CrCl_3_·6H_2_O, thioacetamide (C_2_H_5_NS), and ammonium hydroxide solution dissolved in deionized water as the reactant system, which were heated at various temperatures for 12 h followed by product collection and drying (see Materials and Methods in the online Supplementary file for details).

### Key characterizations

The atomic structures were characterized by using HAADF-STEM and HRTEM. The chemical structures and states were characterized by using XRD, XPS and Raman. The morphologies were studied by using scanning electron microscopy (SEM). The element distribution and ratio were studied by using energy dispersive X-Ray spectroscopy (EDX) and inductively coupled plasma-atomic emission spectrometry (ICP-AES). Thermal properties were studied by using DSC. Electrochemical performances were tested by using 2025-type coin cells, which were assembled mainly by V_1.75_Cr_0.25_S_4_ working electrodes with Super P as the conductive additive and polyvinylidene fluoride as the binder, as well as Li reference electrodes (see Materials and Methods in the online Supplementary file for details).

## Supplementary Material

nwad010_Supplemental_File
