# Supplementary material for: A customized strategy to design intercalation-type Li-free cathodes for all-solid-state batteries
Source: Natl Sci Rev. 2023 Jan 10;10(3):nwad010. doi: 10.1093/nsr/nwad010 (PMC9976772; doi:10.1093/nsr/nwad010)
Supplement: nwad010_Supplemental_File [file nwad010_supplemental_file.pdf]

Supplementary data for

A customized strategy to design intercalation-type Li-free cathodes for all-solid-state batteries

Da Wang[1,7,†], Jia Yu[2,†], Xiaobin Yin[1], Sen Shao[3], Qianqian Li[2], Yanchao Wang[3], Maxim Avdeev[4,5], Liquan Chen[6] and Siqi Shi[1,2,7,*]

[1]School of Materials Science and Engineering, Shanghai University, Shanghai 200444, China;

[2]Materials Genome Institute, Shanghai University, Shanghai 200444, China;

[3]State Key Laboratory of Superhard Materials & International Center for Computational Method and Software, Jilin University, Changchun 130012, China;

[4]Australian Nuclear Science and Technology Organisation, Kirrawee DC, NSW 2232, Australia;

[5]School of Chemistry, University of Sydney, Sydney 2006, Australia;

[6]Institute of Physics, Chinese Academy of Sciences, Beijing 100190, China

[7]Zhejiang Laboratory, Hangzhou 311100, China

[*]**Corresponding author.** Email: sqshi@shu.edu.cn

[†]Equally contributed to this work.

Content

Materials and Methods S3

Section S1: Traditional voltage tuning strategies for intercalation-type cathodes S5

Section S2: Basic structural/electronic properties of MX_2 systems and their Li^+-intercalation properties S6

Section S3: Two improved ligand-field descriptors to balance voltage/phase contradiction under p-type alloying strategy S7

S3.1: $\Delta_{\alpha-\beta}^{\mathrm{CFSS}}$ descriptor for comparing Fermi levels of different phases S7

S3.2: $\mathrm{CFSE}_{\alpha-\beta}$ descriptor for quantifying relative stability of different phases ... S8

Section S4: Selection of $V_xCr_{2-x}S_4$ (x = 0.5, 1, 1.75) using CALYPSO S10

Section S5: Thermodynamic and kinetic stabilities of $V_xCr_{2-x}S_4$ ($0 \leq x \leq 2$) S10

Section S6: Elastic properties of 2H-$V_xCr_{2-x}S_4$ (x = 0.5, 1, 1.75) S11

Section S7: Experimental analysis S12

Section S8: Improved interfacial resistance between 2H-$V_{1.75}Cr_{0.25}S_4$ cathode and typical sulfide solid-state electrolyte S13

Supporting Figures S14

Supporting Tables S34

Appendixes: Optimized geometrical coordinates (POSCAR) of 2H-$V_xCr_{2-x}S_4$ (x = 0.5, 1, 1.75) and $Li_2V_{1.75}Cr_{0.25}S_2/Li_3PS_4$ structures S37

SI References S46

Materials and Methods

Materials: All the reagents used in the experiment were analytical grade and used without further purification. Sodium orthovanadate (Na_3VO_4, 99%, Acros), chromiumchloride hexahydrate ($CrCl_3{\cdot}6H_2O$, 99.9%, Innochem), thioacetamide (TAA, C_2H_5NS, 99%, Aladdin), ammonium hydroxide solution (A.R.) and ethanol (C_2H_5OH, ≥99%). Deionized water was obtained from a Millipore system.

Synthesis of $V_{1.75}Cr_{0.25}S_4$ samples: Flower-like $V_{1.75}Cr_{0.25}S_4$ samples (VCS-450 and VCS-400) were prepared by a facile hydrothermal process. Taking the VCS-400 as an example, firstly, 3.5 mmol of Na_3VO_4, 0.5 mmol $CrCl_3{\cdot}6H_2O$, and 20 mmol thioacetamide (C_2H_5NS) were dissolved in 30 mL deionized water accompanied by string for 1 h. Then 0.8 mL ammonium hydroxide solution was added to the above solution and stirred again for 15 min to form a homogenous solution. Next, the solution was transferred to a Teflon-lined stainless-steel autoclave and heated at 400 K for 12 h. After natural cooling, the precipitate was collected by centrifugation and washed with deionized water and ethanol for several times. Finally, the collected product was dried in a vacuum oven at 333 K overnight to obtain VCS-400. VCS-450 was prepared by a similar method except for a heating temperature of 450 K. VS_2 was prepared at 450 K in absence of $CrCl_3{\cdot}6H_2O$.

Characterizations: The chemical structures of the as-synthesized samples were characterized using an X-ray diffractometer (XRD, Panalytical Empyrean) with Cu Kα radiation at a voltage of 60 kV. The morphologies were studied using a scanning electron microscope (SEM, Hitachi/SU8230) at a voltage of 5 kV with an Oxford XMax^N energy-dispersive X-ray spectroscopy (EDX) detector. The atomic arrangement of 2H and 1T phases were characterized using an environmental spherical aberration corrected transmission electron microscope (TEM, ThermoFisher/FEI THEMIS ETEM G3) at a voltage of 200 kV. The chemical states were obtained using an X-ray photoelectron spectrometer (XPS, PerkinElmer/PHI 5600) with a Mg-Ka light source. The metal-sulfur vibration mode was obtained from a microscopic Raman spectroscopy system (Raman, Renishaw inVia) using a 532 nm laser. Heat flux were obtained using a differential scanning calorimeter (DSC, Netzsch/214 Polyma) with Ar atmosphere and a heating rate of 5 K min^{-1}.

Electrochemical tests: The working electrode was composed of 70 wt.% of VCS-400 (or VCS-450) sample as active material, 20 wt.% of Super P as conductive additive, 10 wt.% of polyvinylidene fluoride (PVDF) as binder. Using N-methyl-2-pyrrolidone (NMP) as solvent, the mixed slurry was pasted onto an aluminum foil followed by drying at 80 °C for 12 h in vacuum oven. Then the electrode was cut into 10 mm diameter round pieces for use. The mass loading of active materials is ~1.6 mg cm^{-2}. Polypropylene and Li foil were employed as the separator and reference electrode, respectively. The electrolyte was 1.0 M lithium hexafluorophosphate ($LiPF_6$) dissolved in in ethylene carbonate (EC) + Ethyl Methyl Carbonate (EMC) + dimethyl carbonate (DMC) (weight ratio of 1: 1: 1). The 2025-type coin cells were assembled in an argon-filled glove-box. Galvanostatic charge-discharge cycling at 0.1 C and 1.7–2.8 V voltage range was tested using on a NEWARE battery testing system. The galvanostatic intermittent titration technique (GITT) test was to discharge the cell at 0.02 C for 15 seconds followed by relaxation for 15 min. For fabricating all-solid-state lithium batteries, VCS-400 (or VCS-450, VS_2, TiS_2), Li_3PS_4 (LPS) solid electrolytes, and Super P were mixed homogeneously by manual grinding in a weight ratio of 45:45:10; then solid electrolytes were pressed in a poly(tetrafluoroethylene) mold under 200 MPa, and the cathode was distributed on the LPS uniformly, and metal lithium foil was pressed on the opposite side.

Section S1: Traditional voltage tuning strategies for intercalation-type cathodes

In this part, we summarize the electrochemical potential tuning strategies commonly used in the transition-metal-based cathodes for lithium-ion batteries (LIBs), as shown in Fig. S2. Essentially, all these strategies are motivated by changing the ionic/covalent nature of the metal and ligand bond (M–X) that controls the Fermi level in systems, *viz.*, the ionic/covalent change of the M–X bond will govern the quantum mechanical repulsion between bonding and antibonding orbitals, shifting the position of the valence-band top with respect to the Li/Li^+ energy level, and therefore changing the potential required for the removal of one electron (Fig. S2A). We note that these potential tuning strategies can be divided into the following two types (Fig. S2B): (i) The strategies that directly impact the ionic/covalent nature of M–X bond, such as electronegativity and transition-metal coordination tuning strategies. Previous studies suggested that the higher electrochemical potential of $LiCoPO_4$ cathode (~4.8 V) than $LiFePO_4$ cathode (~3.4 V) is primarily caused by the higher electronegativity of Co (in $LiCoPO_4$, 1.9) than Fe (in $LiFePO_4$, 1.8) [1, 2]. Besides, Gutierrez *et al.* [3] proposed that with the increase of coordination number, the steric hindrance increases, which leads to the more stable M–X bond, thus providing a higher voltage. As a result, the $LiFePO_4$ system with a 6-coordinate octahedral structure exhibits the highest potential (3.4 V) than $LiFeBO_3$ (3 V) with 5-coordinate triangular-bipyramid structure as well as Li_2FeSiO_4 (2.8 V) with 4-coordinate tetrahedron structure. (ii) The strategies that impact ionic/covalent nature of M–X bond by the indirect primary/secondary inductive effect. The primary inductive effect refers to the case where the charge density on M–X bond is separated by the counter cation, thus reducing the covalent of the M–X bond and increasing the potential, and the secondary inductive effect corresponds to the case where the charge density on M–X bond is partitioned by the neighboring Li atoms, thereby increasing the potential of system. For example, Manthiram *et al.* [4, 5] indicated that the inductive effect elicited by Si–O bond leads to the weakening of Fe–O bond in $Fe_2(SO_4)_3$, resulting in a significant potential-increasing of $Fe_2(SO_4)_3$ (~3.6 V) compared to the pristine Fe_2O_3 cathode (~2.5 V). It is important to emphasize that ionic/covalent nature of above M–X bond is usually not determined by a single factor, even so, based on the above discussions, we conclude that all these general strategies cannot achieve a significant increase of Li^+-interaction potential.

Section S2: Basic structural/electronic properties of MX_2 systems and their Li^+-intercalation properties

Group-VIB MX_2 mainly crystallize in 2H (space group $P6_3/mmc$, #194) and 1T (space group $P\overline{3}m1$, #164) polymorphs. Other derived phases, such as 1T' and 3R possess similar intercalation environments for Li^+ to that of the 1T or 2H phase, thus only these two phases are considered in this work. Examination of the dependence of voltage on W_{fill} for Li^+-intercalation in Group-VIB MX_2 shows that both MS_2 and MSe_2 have different slopes and intercepts within their individual linear relations. This is because the total energy calculated from first-principles calculation contains three parts (total energy of occupied electronic eigenstates, ion-ion Coulomb energy, and Hartree energy), which is expressed as: $E_{total} = \int \rho(E)dE + E_{ion-ion} - \frac{e^2}{2}\int dr \int dr' \frac{\rho(r)\rho(r')}{|r-r'|}$. The defined W_{fill} only takes into account the energy change caused by introduced Li-2s in conduction band under the rigid-band approximation, contributing to the first term in the above equation. Other possible contributions to the voltage, such as changes in $E_{ion-ion}$ as well as Hartree energy are not considered, resulting in the linear dependence deviation between MS_2 and MSe_2 compounds. These influences can easily be confirmed by the volume expansion values of the Group-VIB MX_2 intercalated compounds, *e.g.*, for MS_2 compounds, the smaller ionic radius and lower energy level of sulfur lead to greater volume expansion (11.5%–17.5%) than that of MSe_2 (10%–16.5%) during Li-ion intercalation process.

Section S3: Two improved ligand-field descriptors to balance voltage/phase contradiction under p-type alloying strategy

S3.1: $\Delta_{\alpha-\beta}^{\mathrm{CFSS}}$ descriptor for comparing Fermi levels of different phases

The Fermi level position of electrode is mainly determined by its crystal field splitting strength (CFSS), which is affected by two aspects: (i) TM-d orbitals splitting strength, Dq= $\frac{Z_L e^2 \langle r^4 \rangle}{6R^5}$, where Z_L is the charge on ligands, e is the electron charge, R is the average interatomic distance between M and ligand. $\langle r^4 \rangle$ is the mean radial distance between electrons and nucleus exponentiated to the fourth power. (ii) Splitting coefficient of d orbitals that determines the Fermi level, *n*. Under the action of perturbation potential, the splitting of d-orbital directly affects the molecular orbital level and eventually determines Fermi level of systems (Table S3).

Take the common octahedron ligand field as an example, the d orbital is divided into two different symmetries ($\mathrm{e_g}$ and $\mathrm{t_{2g}}$) due to the influence of the perturbation potential, and the energy difference between the two orbitals is $\Delta_0 = \mathrm{E}(\mathrm{e_g}) - \mathrm{E}(\mathrm{t_{2g}}) = 10\mathrm{Dq}$. It is suggested that the strength of Dq can be described by [6]: Dq= $\frac{\langle r^4 \rangle Z_L e^2}{6R^5}$. It is seen that the 10Dq split increases with the increasing of the radial integral $\langle r^4 \rangle$ of central atom. For different central ions, the calculation method of $\langle r^4 \rangle$ are different. It is believed that the value of $\langle r^4 \rangle$ is nearly constant for the same transition metal ion in the same ligand environments. However, for this simple processing method, the relationship between the crystal field splitting and the coordination field environment cannot be quantitatively reflected. Then Shi *et al.* [7] studied the quantitative relationship between the crystal splits of Ce^{3+} and Eu^{2+} in halide crystals and environmental factors. By comparing the calculated data with theoretical formula, when the central ion is the same, the ion radius r_i can be used instead of $\langle r^4 \rangle$. By using the ion radius r_i instead of $\langle r^4 \rangle$, it is possible to quantitatively compare the degree of splitting of transition metal central atoms in the same period.

Here we propose a descriptor to compare the Fermi levels of specified α and β phases:

$$\Delta_{\alpha-\beta}^{\mathrm{CFSS}} \left(= n_\alpha \frac{Z_L e^2 {r_i}^4}{6R_\alpha^5} - n_\beta \frac{Z_L e^2 {r_i}^4}{6R_\beta^5}\right) \tag{S1}$$

where n_α and n_β indicate the M-d orbital splitting coefficients that determine α and β phases Fermi-level, respectively.

S3.2: $\mathrm{CFSE}_{\alpha-\beta}$ descriptor for quantifying relative stability of different phases

The phase stability is closely related to the crystal field splitting of transition-metal center in systems, which contribute not only to the lowering of crystal field stabilization energy (CFSE), but also to the reduction of electronic configurational entropy (S_{CFS}), by removing orbital degeneracy: $\mathrm{G_{CFS}} = -\mathrm{CFSE} - \mathrm{T} \times \mathrm{S_{CFS}}$ [8, 9]. Considering the contribution of entropy in the ground state (0 K) negligible, the relative energy stability of α and β phases ($\mathrm{G_{CFS_{\alpha-\beta}}}$) can be approximated by crystal field stabilization energy difference ($\mathrm{CFSE}_{\alpha-\beta}$). It is suggested that CFSE is determined by three facts: (i) The tendency of electrons to occupy the lowest energy orbital in ligand field, which is depends on Dq. It determines the degenerate state and energy level position of the d orbital. (ii) The pairing energy (P) generated by the electrons entering the d orbital in different spin states. (iii) The degenerate splitting caused by the local structural distortion, *e.g.*, Jahn-Teller effect. Eventually, it can be presented as: $\mathrm{CFSE} = \left((n_1 N_x + n_2 N_y + \cdots \cdots)\mathrm{Dq} + m\mathrm{P} + \frac{2(\delta+\sigma)}{3}\right)$, where n_1 (or n_2) represent the symmetry coefficients of different orbitals, as summarized in Table S3, and N_x, N_y, N_z are the number of electrons in the above respective orbitals. The term $\frac{2(\delta+\sigma)}{3}$ is the extra stabilization enthalpy gained by M^{4+} as a result of the MX_6 distortion, which can be ignored here in MX_2 with the same structure. Notably, *m*-factor in front of pairing energy depends on the number of forced pairing electrons (See Table S4 and Table S5 for different values of ligand-field systems, respectively). Here we take octahedron ligand-field system as an example to illustrate the change rule of the pairing energy with the number of d electrons. If there are only two or three d electrons, both the above-mentioned tendencies can be satisfied simultaneously by placing the electrons in different t_{2g} orbitals with their spins parallel. However, when there are more than three d electrons this is no longer possible. For a d^4 system, occupancy of the t_{2g} and e_g orbitals will be different in a strong field and weak field complex. Fig. S7 shows distribution of d^4 electron in the strong field and weak field complex. In the strong field complex $\Delta_0 > \mathrm{P}$, so the fourth electron pairs up in the $\mathrm{t_{2g}}$ orbital and gives the electron configuration of the metal as $\mathrm{t_{2g}^4 e_g^0}$. In the weak field complex $\Delta_0 < \mathrm{P}$, so the fourth electron occupies $\mathrm{e_g}$ orbital and gives the electron configuration of the metal as $\mathrm{t_{2g}^3 e_g^1}$. If there are 4–7 d-electrons, there are different cases of putting as many electrons into the low-energy $\mathrm{t_{2g}}$ orbital, or distributing them so as to maintain a maximum number of parallel spins. Each electron in $\mathrm{t_{2g}}$ orbital lowers the energy of the system by 0.4Dq, whereas each

electron in an e_g orbital raises the energy by 0.6Dq. In addition, each electron pair forced to be paired in the same orbital raises the energy of the system by the pairing energy P. For d^4-d^7 configurations, if Dq < P, the system is more stable if the electrons occupy the e_g orbitals rather than being paired in the t_{2g} orbitals, giving rise to high-spin complexes. If Dq > P, a low-spin complex results in which the electrons are paired in t_{2g} orbitals rather than occupying higher-energy e_g orbitals. For Group-VB/VIB MX_2 systems considered in our work, the electronic configuration is d^1/d^2, as illustrated in the small inset in Fig. 1D. Thus, no paired electrons are formed after the d electrons split, resulting in no paired energy being produced in these systems. As a result, we only need to consider the splitting energy to measure crystal field stabilization energy, CFSE ($= \left(n_1 N_x + n_2 N_y + \cdots\right)$Dq).

Notably, for compounds containing different M element periods, the above method cannot be used. This is because the characteristics of the d orbital itself, such as electron shell and nuclear charge number, must be considered. Theoretically, Hamiltonian of metal ion is often written as a sum of the free metal ion Hamiltonian and a single electron crystal field Hamiltonian containing the so called A_{kq} crystal field parameters. The Hamiltonian of the crystal field in this model can be written as $H' = \sum_{i,j} \frac{Z_j e^2}{|r_i - R_j|} = \sum_{k,q} A_{kq} \langle r^k \rangle C_q^k$, where $\langle r^k \rangle$ is the radial integral of central atom, A_{kq} is the crystal field parameter depending only on the host crystals, independent of central ions. C_q^k is the coordinate parameter of electrons, which can be calculated based on angle coordinates of electrons. Obviously, the crystal splitting is dominated by two parts: one is the central-ion; the other is the ligand environment [10]. We establish an improved crystal field strength descriptor, which divides Dq as a product of a function f of ligands and g of central-ion: $\mathrm{Dq} = \mathrm{g(central\ ion)} \times \mathrm{f(ligands)} = \mathrm{g}' \times \frac{Z_L e^2 {r_i}^4}{6R^5}$ (where g′ represents a spectrochemical series of central ions for the same ligand, showing a strict arrangement according to an increasing number of the transition group $3d^n < 4d^n < 5d^n$ (with relative values of the functions 1: 1.45: 1.75) or an increasing oxidation numbers +2 < +3 < +4 (with relative values of g′ approximately 1: 1.6: 1.9). Then we can directly calculate the crystal field stabilization energy difference between specified α and β phases:

$$\mathrm{CFSE}_{\alpha-\beta} = \left(n_1 N_x + n_2 N_y + \cdots\cdots\right)_\alpha \times \left(\mathrm{g}' \times \frac{Z_L e^2 {r_i}^4}{6R^5}\right)_\alpha - \left(n_1 N_x + n_2 N_y + \cdots\cdots\right)_\beta \times \left(\mathrm{g}' \times \frac{Z_L e^2 {r_i}^4}{6R^5}\right)_\beta \quad \text{(S2)}$$

Section S4: Selection of $V_xCr_{2-x}S_4$ (*x* = 0.5, 1, 1.75) using CALYPSO

In this work, the particle swarm optimization algorithm implemented in the CALYPSO (Crystal structure AnaLYsis by Particle Swarm Optimization) method [11, 12] was used for the searching of stable $V_xCr_{2-x}S_4$ ($0 \leq x \leq 2$) compounds. The effectiveness and the efficiency of this crystal search method have been proven by many well-studied systems, including elements and binary and ternary compounds [13]. Any new materials predicted by CALYPSO have been also experimentally confirmed [14, 15]. With the aid of this powerful tool, we obtained the most stable structures of the above selected compounds, as illustrated in Fig. 3A.

Section S5: Thermodynamic and kinetic stabilities of $V_xCr_{2-x}S_4$ ($0 \leq x \leq 2$)

We search structures on stoichiometry $V_xCr_{2-x}S_4$ ($0 \leq x \leq 2$). During the structure searching, unit cells are limited up to 4 formulas for each stoichiometry. Both the generation sizes and the number of generations were set to 30 for getting a convergent result. The structures with lowest energy and reasonable coordination geometry are shown in the Fig. 3A of main text.

To construct phase diagrams, we extract all structural prototypes and energies in the V–Cr–S ternary system in the Inorganic Crystal Structure Database (ICSD) and Materials Project (MP) Database (Table S6). All energies are presented as per formula unit (*f.u.*) formation energies, E_f, from the elements, Cr, V and S. The structures and energies of 2H-$V_{0.5}Cr_{1.5}S_4$, 2H-$VCrS_4$ and 2H-$V_{1.75}Cr_{0.25}S_4$ studied in this paper are obtained by the first-principles calculations, which are performed with VASP.

The thermodynamic stabilities of 2H-$V_{0.5}Cr_{1.5}S_4$, 2H-$VCrS_4$ and 2H-$V_{1.75}Cr_{0.25}S_4$ are described in the main text. It is suggested that all 2H-$V_{0.5}Cr_{1.5}S_4$, 2H-$VCrS_4$ and 2H-$V_{1.75}Cr_{0.25}S_4$ are located at the formation energy convex hull, indicating their superior thermodynamic stability (Fig. S8). Besides, for kinetic stability, in Fig. S9, no imaginary frequency has been found in the phonon dispersion curves of $V_{0.5}Cr_{1.5}S_4$, $VCrS_4$ and $V_{1.75}Cr_{0.25}S_4$, showing their good kinetic stability. Furthermore, we performed the MD simulation of $V_xCr_{2-x}S_4$ ($0 \leq x \leq 2$) at 400 K (Fig. S10). The results show that these structures will maintain well at the cell operating temperatures.

It should be noted that the V–Cr–S phase diagram (T = 0 K, Fig. S8) does not consider the effect of temperature, which may affect the relative phase stability of 2H- *vs.* 1T-$V_xCr_{2-x}S_4$ phases due to the entropic contributions on free energy at elevated temperature. By calculating the phonon spectrums and vibrational entropies of 2H/1T-$V_{0.5}Cr_{1.5}S_4$, 2H/1T-$VCrS_4$ and 2H/1T-$V_{1.75}Cr_{0.25}S_4$ using harmonic approximation (Fig. S9), we obtain the temperature dependence of the free energies between two phases (ΔF_{1T-2H}) in these three systems (Fig. 3B).

Section S6: Elastic properties of 2H-$V_xCr_{2-x}S_4$ (x = 0.5, 1, 1.75)

The elastic moduli, such as bulk modulus B and shear modulus G of 2H-$V_xCr_{2-x}S_4$ (x = 0.5, 1, 1.75) systems were derived based on the Voigt-Reuss-Hill (V-R-H) approximation (Table S8) [16]. The elastic tensor, C_{ij} gives the upper limit of B and G.

$$B_v = \frac{(C_{11}+C_{22}+C_{33}) + 2(C_{12}+C_{23}+C_{31})}{9} \quad \text{(S3)}$$

$$G_v = \frac{(C_{11}+C_{22}+C_{33})-(C_{12}+C_{23}+C_{31}) + 3(C_{44}+C_{55}+C_{66})}{15} \quad \text{(S4)}$$

In the Reuss approximation, the compliance tensor, $s_{ij}(= C_{ij}^{-1})$ is based on uniform stress, leading the lower boundary limit.

$$B_R = \frac{1}{(s_{11}+s_{22}+s_{33})+2(s_{12}+s_{23}+s_{31})} \quad \text{(S5)}$$

$$G_R = \frac{15}{4(s_{11}+s_{22}+s_{33})-4(s_{12}+s_{23}+s_{31})+3(s_{44}+s_{55}+s_{66})} \quad \text{(S6)}$$

In Hill approximation, arithmetic average of Voigt and Reuss boundary limits is: $B = \frac{B_V+B_R}{2}$, $G = \frac{G_V+G_R}{2}$. In addition, Young's moduli E and Poisson's ration v is: $E = \frac{9BG}{(3B+G)}$, $\nu = \frac{3B-2G}{2(3B+G)}$. We also applied the Born elastic stability criterion to check the mechanically stable of 2H-$V_xCr_{2-x}S_4$ under zero pressure. This criterion in harmonic approximation states that for a mechanically stable compound, the relevant elastic tensor must be positive definite. The calculated elastic constants demonstrate that all 2H-phase $V_{0.5}Cr_{1.5}S_4$, $VCrS_4$ and $V_{1.75}Cr_{0.25}S_4$ are mechanically stable (Table S8).

Section S7: Experimental analysis

A) SEM observation:

The morphologies of VCS-400 and VCS-450 were observed by scanning electron microscopy (SEM). In both low and high magnifications, the VCS-400 has 3D flower-like microstructures assembled with dozens of 2D nanosheets, with a lateral size of 2–4 μm as well as good uniformity (Fig. S19A, B). These nanosheets with smooth surface and regular edges connect to each other to form 3D hierarchical structures. The VCS-450 shows similar microstructure with similar size (Fig. S19C, D).

B) Element analysis:

Energy dispersive X-ray spectroscopy (EDS) mapping results confirmed a uniform distribution of V, Cr, and S elements (Fig. S19E). Furthermore, inductively coupled plasma atomic emission spectrometry (ICP-AES) showed that the Cr/V element ratios agreed with the theoretical values, evidencing a successful doping of Cr into V-based matrix.

C) XRD analysis:

The characteristic (001) diffraction peaks at 15.2° for both the VCS-400 and VCS-450 samples indicate a stacked lamellar structure. Especially, the slightly wider peaks of VCS-400 mean lower crystallinity and smaller grain size, being consistent with its lower synthesis temperature [17].

D) Galvanostatic discharge-charge test:

During the discharge-charge cycles, both VCS-400 and VCS-450 delivered initial discharge capacities very close to the theoretical value of ~230 mAh g^{-1}, and maintained stability with a high Coulombic efficiency (CE) after 50 cycles.

E) Cross-sectional SEM observation:

Based on the average thickness of electrode pieces, the volume expansion degree of VCS-400 after initial lithiation process (to 1.7 V) was estimated to be ∼4.1% (15.1 μm) as compared with the pristine state (14.5 μm), rendering a considerable structural stability that agreed with the above discharge-charge cycling test. Similarly, the volume expansion degree of VCS-450 after initial lithiation process was estimated to be ∼4.7%.

F) XPS analysis:

For the pristine VCS-400 samples, the two peaks at 517.2 and 524.5 eV correspond to the spin-orbit splitting of V $2p_{3/2}$ and V $2p_{1/2}$ of V^{4+} cations, respectively, when two peaks at 516.0 and 523.2 eV correspond to the spin-orbit splitting of V $2p_{3/2}$ and V $2p_{1/2}$ of V^{3+} cations, respectively [18]. Peak-fitting analysis of S 2p XPS confirmed the presence of S $2p_{3/2}$ and S $2p_{1/2}$ peaks of S^{-} and S^{2-} [19]. When it was discharged to 1.7 V, the peak area ratio of the S^{2-} were obviously enhanced, and the whole peak shifted to lower binding energies as indicated in Fig. 4H. Similarly, the decrease of the chemical valence of V is also observed in Fig. 4G.

Section S8: Improved interfacial resistance between 2H-$V_{1.75}Cr_{0.25}S_4$ cathode and typical sulfide solid-state electrolyte

Here we systematically calculated and compared the space charge layer effect at the interface between oxide cathode/sulfide solid-state electrolyte ($LiCoO_2$ (LCO)/Li_3PS_4 (LPS)) and full-discharged sulfide cathode/sulfide electrolyte ($Li_2V_{1.75}Cr_{0.25}S_4$ (LVCS)/LPS) in the equilibrium and initial charging states. We selected LCO as cathode for the present investigation because it is the most widely used cathode material for LIBs, and some studies on the interface are available [20]. Besides, LPS is adopted as a typical example of sulfide electrolyte because it has an ionic conductivity comparable to that of organic liquid electrolytes (~10^{-4} S cm^{-1}) [21]. In addition, the crystal structure of LPS is simpler than other sulfide electrolytes such as $Li_7P_3S_{12}$ or $Li_{10}GeP_2S_{12}$, which is important for controlling the atomic size of the interface structure as well as the computational efficiency [22, 23]. We build a slab model of LVCS/LPS and calculate the Li vacancy formation energy in the interface model. LVCS (001), LCO (110) [24] and LPS (010) [25] are selected as the initial surfaces when building slab model, although these surfaces are not necessarily the most stable, they are most likely the conduction path of Li^{+} [26]. In order to eliminate the influence of interface stress and lattice periodicity, we guarantee the interface mismatch (< 10%) and add 1.5 nm vacuum layer to supercell. Considering that the vacancy formation energy of Li atom (E_v) with respect to Li metal can be regarded as Li chemical-potential, the detailed Li^{+} migration behavior under equilibrium and at the initial stage of charging are discussed based on the present calculation results. The relaxed structure shows that Li ions tend to move from LVCS side to LPS side. According to the calculation results, the space charge layer effect also exists in LVCS/LPS at equilibrium state, but it is weaker than LCO/LPS (the difference of Li^{+} concentration on both sides of the LVCS/LPS interface is lower, Fig. S32). As a result, $Li_2V_{1.75}Cr_{0.25}S_4$/Li_3PS_4 has an improved interfacial resistance than $LiCoO_2$/Li_3PS_4, and its interface is relatively stable.

Supporting Figures

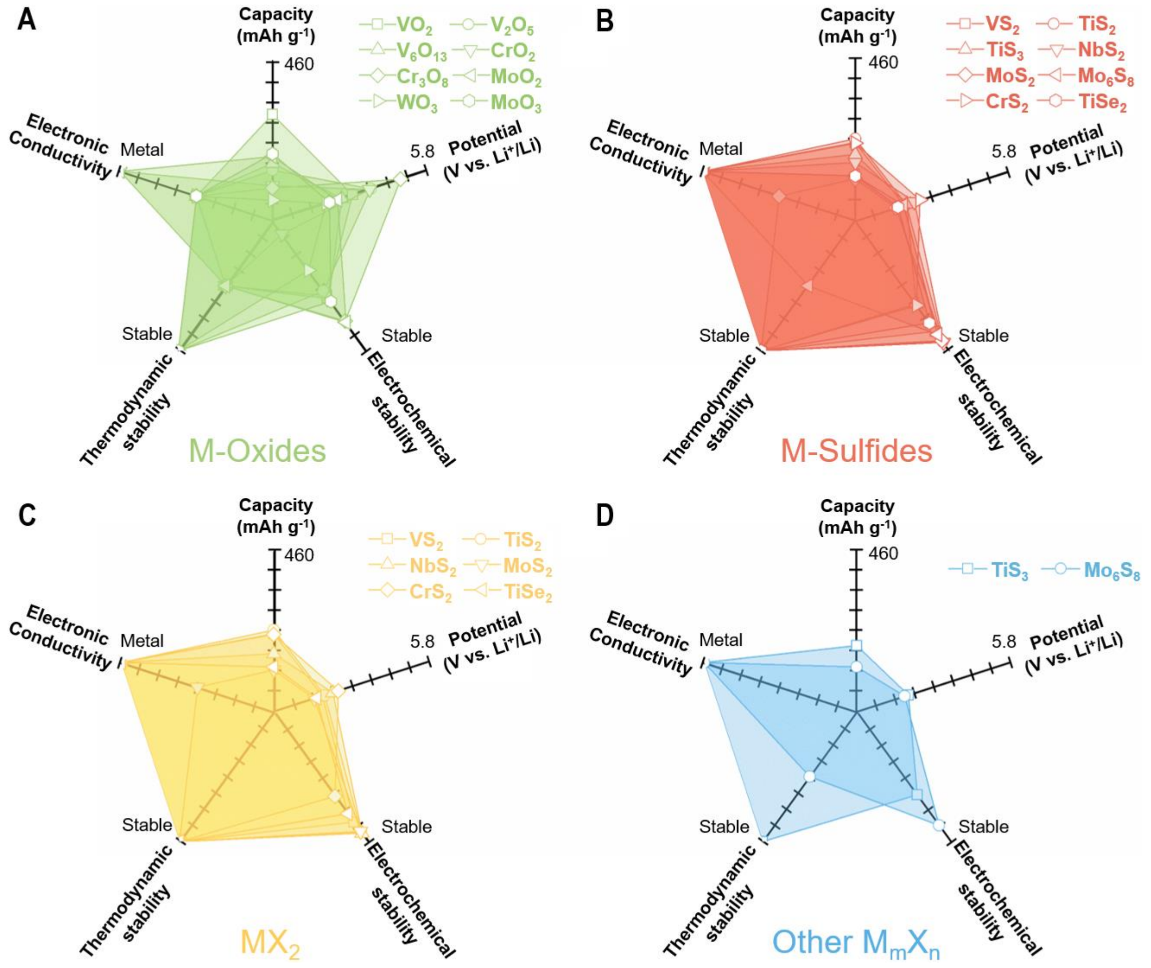

Figure S1. Radar plots of the electrochemical properties of different Li-free cathodes: (A) transition-metal oxides (M-Oxides), (B) transition-metal sulfides (M-Sulfides), (C) transition metal dichalcogenides (MX_2) and (D) other transition metal sulfides (M_mX_n). The mass specific capacity, electrochemical potential, thermodynamic stability, electronic conductivity, and electrochemical stability (or reversibility) data are obtained from Material Project (MP) database [27] or experimental results [28-39]. Electrochemical stability is measured by the remaining capacity ratio after a certain number of cycles (10–50 cycles) during a constant charge/discharge rate in the range of 0.1 C–0.4 C, where "Stable" represents no attenuation (0%) under ideal conditions. Thermodynamic stability can be divided into "Stable" and "Metastable" states according to the calculated phase diagram given in the MP database, and electronic conductivity can also be divided into "Metal" and "Semiconductor" based on the calculated band gaps given in the database.

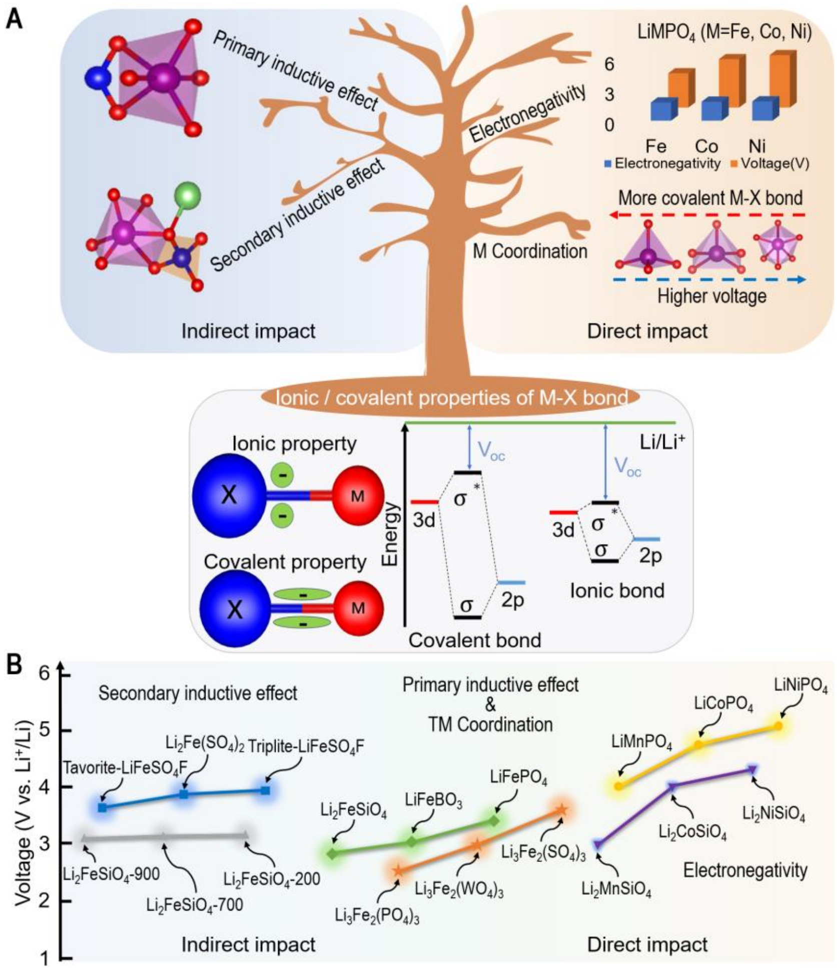

Figure S2. Traditional voltage regulation strategies for Li-ion batteries. (A) A schematic of the relationship between electronegativity, TM coordination and induction effect, which affect the ion/covalent properties of the M–X bond; (B) Data for the electrochemical potential variation trend of $LiMPO_4$ (M = Mn, Co, Ni), Li_2MSiO_4 (M = Mn, Co, Ni), $LiFePO_4$, $LiFeBO_3$, Li_2FeSiO_4, $Li_3Fe_2(XO_4)_3$ (X = P, W, S), Li_2FeSiO_4@X (X = 900 K, 700 K, 200 K), tavorite-$LiFeSO_4F$, $Li_2Fe(SO_4)_2$ and triplite-$LiFeSO_4F$ are collected from Refs. [2, 3, 40, 41].

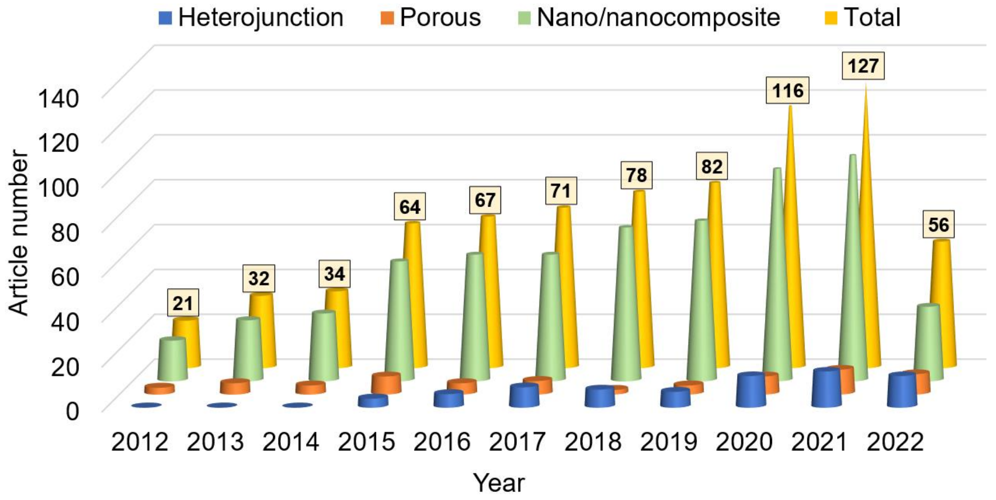

Figure S3. The improvement of capacity, electronic conductivity and cycling performance of MX_2 materials (X = S, Se) by different structural/morphological modification technologies (*e.g.*, heterojunction, porous and nanocomposite) in the past decade (2012-2022).

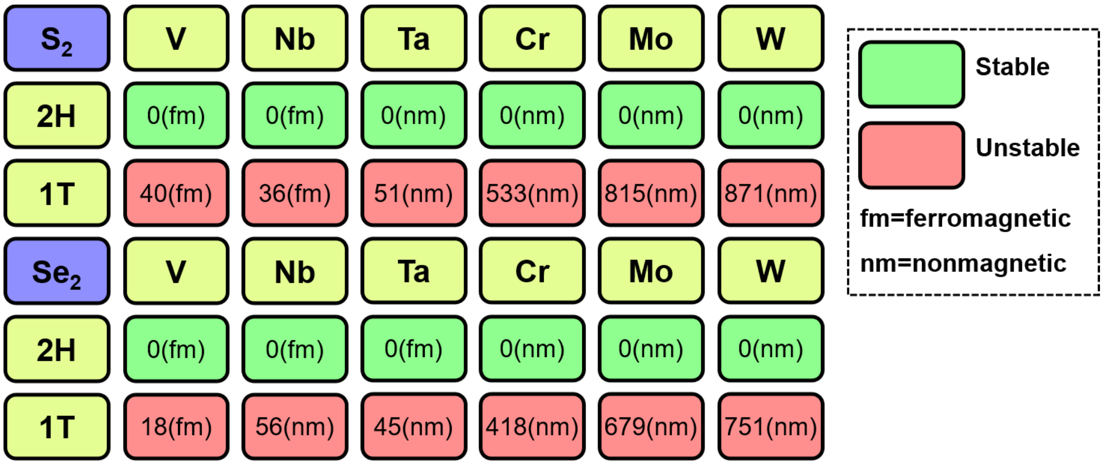

Figure S4. Relative total energy between 1T and 2H phases (2H phase is used as reference, so $\Delta E = E_{1T} - E_{2H}$, meV) for Group-VB (V, Nb, Ta)/VIB (Cr, Mo, W) MX_2 (X = S, Se). The magnetic ordering is also shown in parentheses: nonmagnetic (nm) and ferromagnetic (fm). Classification of stable phases according to ΔE: stable (green) and unstable (red, and the predicted metastable energy values are shown in meV per MX_2 unit).

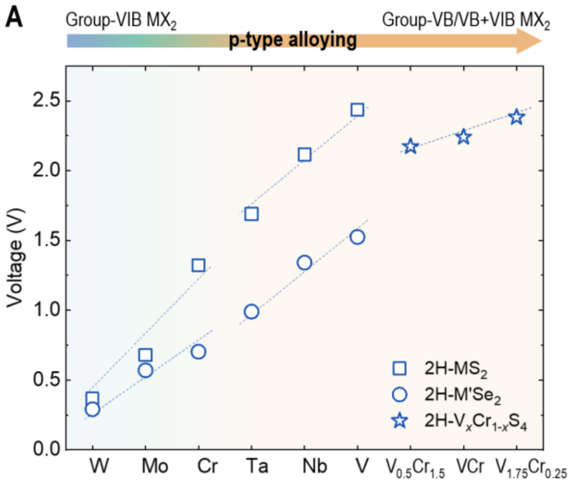

Figure S5. The average Li^+-intercalation voltages of Group-VB MX_2 (M = W, Mo, Cr; X = S, Se), Group-VIB MX_2 (M = Ta, Nb, V; X = S, Se), and Group-VB/VIB $V_xCr_{1-x}S_4$ (x = 0.5, 1.0, 1.75) with (A) 2H and (B) 1T phase structures, respectively.

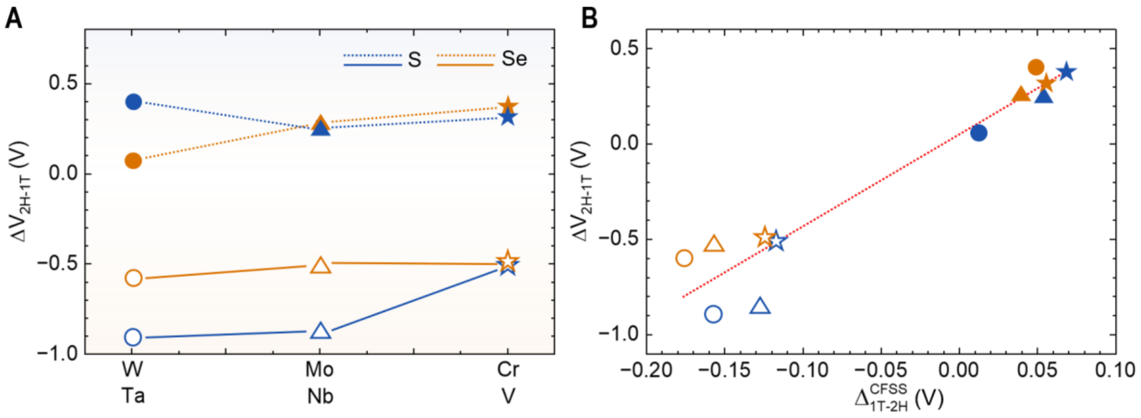

Figure S6. (A) Average Li^+-intercalation voltage difference between 2H- and 1T-phases (ΔV_{1T-2H}) of Group-VIB/VB MX_2 with the change of M-element period. (B) ΔV_{1T-2H} as a function of Δ^{CFSS}_{1T-2H} for Group-VIB/VB MX_2. The voltages of Group VB MX_2 with 2H phase structures are higher than that with 1T phase structures, and the trend is reversed for Group VIB MX_2 electrodes.

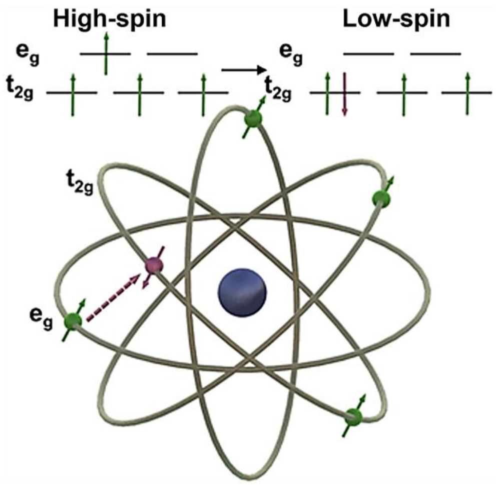

Figure S7. Crystal field splitting for octahedral low-spin and high-spin d^6 complexes.

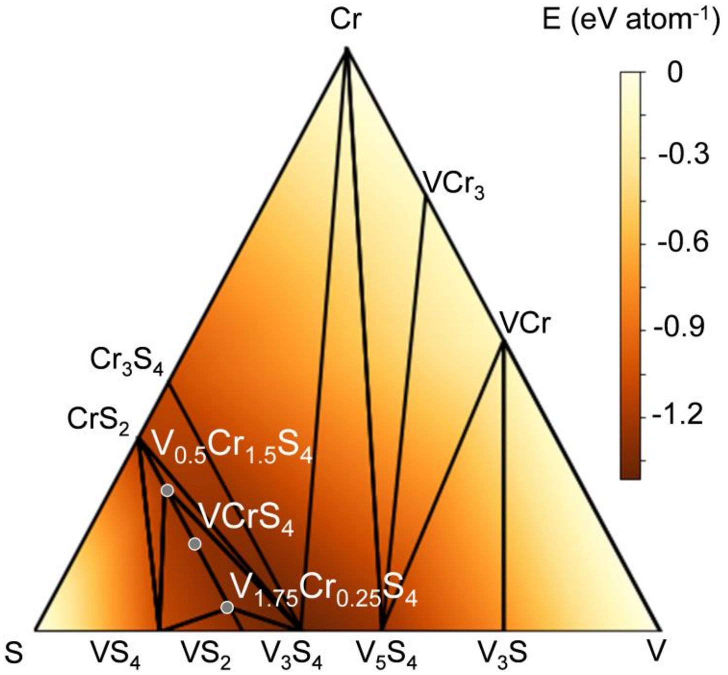

Figure S8. Grand potential phase diagrams for V–Cr–S systems under 0 K. All 2H-$V_{0.5}Cr_{1.5}S_4$, 2H-$VCrS_4$ and 2H-$V_{1.75}Cr_{0.25}S_4$ are located at the formation energy convex hull, indicating their superior thermodynamic stability.

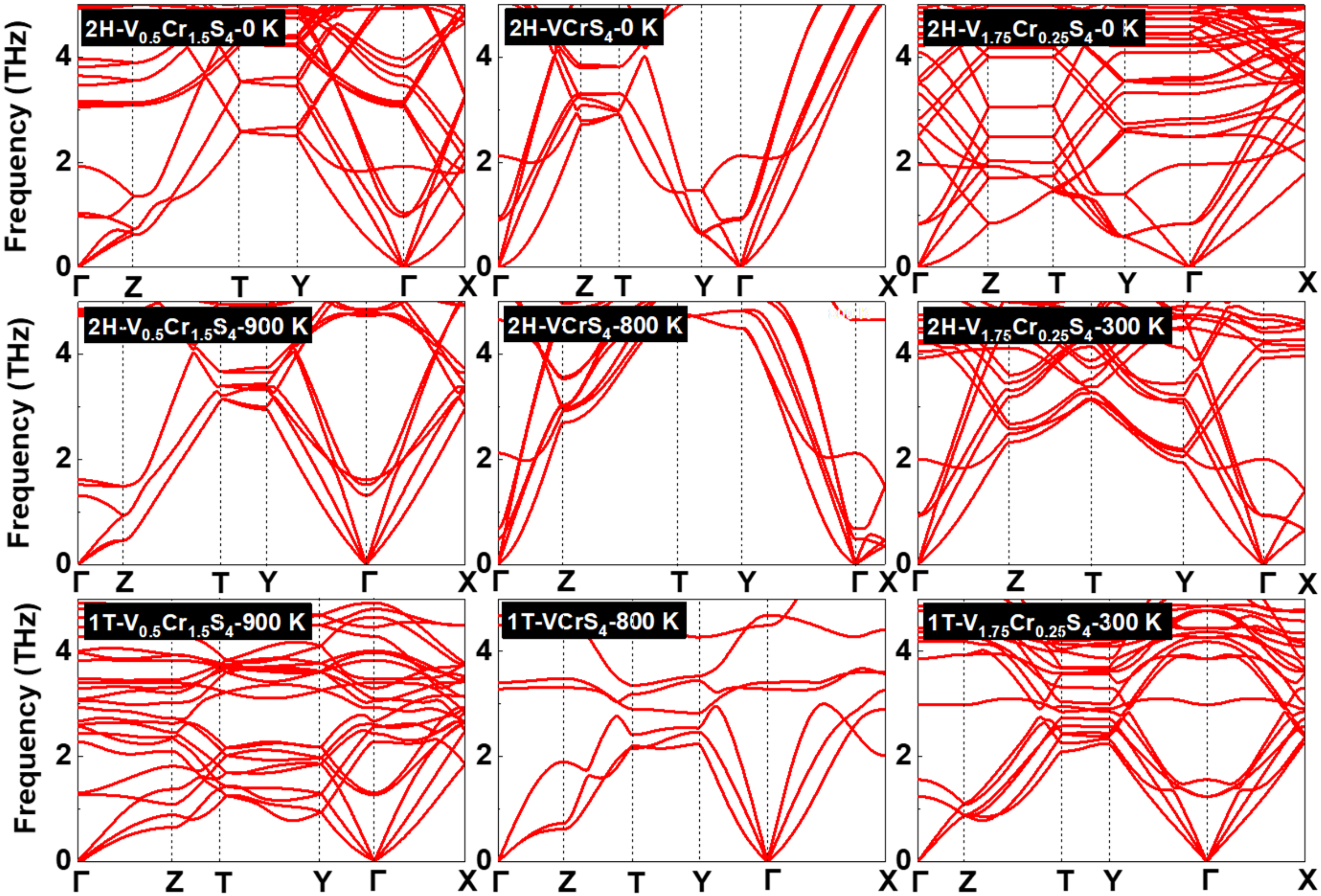

Figure S9. Phonon dispersion of the ground-state 2H-$V_xCr_{2-x}S_4$ (x = 0.5, 1, 1.75), and phonon dispersion of 1T/2H-$V_xCr_{2-x}S_4$ (x = 0.5, 1, 1.75) considering temperature. It shows that 1T-phase $V_{0.5}Cr_{1.5}S_4$, $VCrS_4$ and $V_{1.75}Cr_{0.25}S_4$ are stable at 900 K, 800 K and 300 K, respectively.

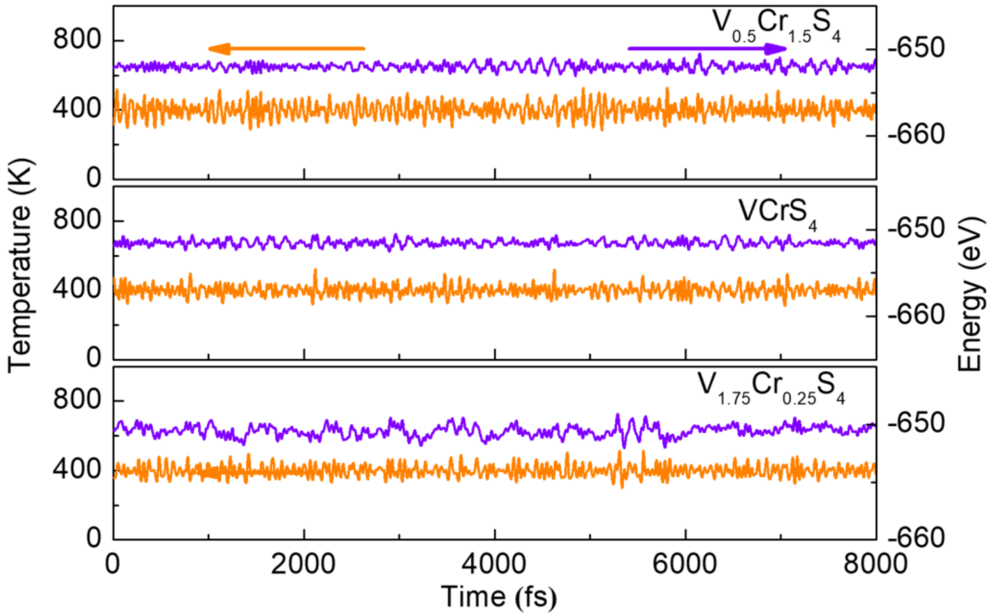

Figure S10. Total energy and temperature fluctuation of a 2 × 1 × 1, 1 × 4 × 2, 1 × 2 × 1 supercell (96 atoms) for $V_{0.5}Cr_{1.5}S_4$, $VCrS_4$ and $V_{1.75}Cr_{0.25}S_4$ at 400 K with a time of 8000 fs. Here the cutoff energy is set to 400 eV, and only Gamma point was used in the Brillouin zone sampling. All structures remain stable at the cell operating temperatures (< 400 K).

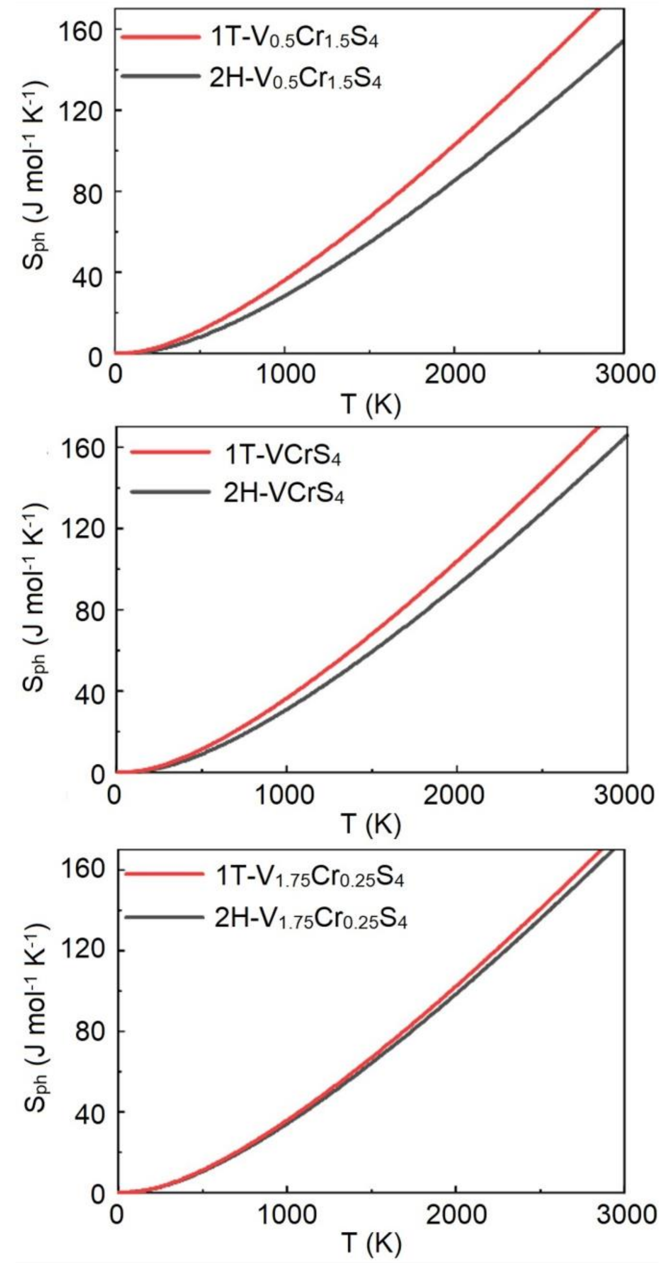

Figure S11. The calculated vibration entropy (S_{ph}) of 1T/2H-$V_xCr_{2-x}S_4$ (x = 0.5, 1, 1.75) systems.

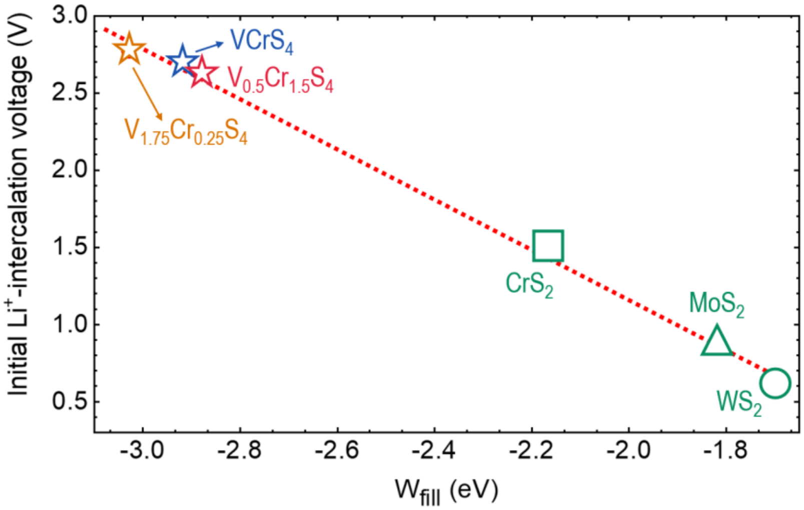

Figure S12. The initial Li^+-intercalation voltage of 2H-$Li_{0.0625}MS_2$ (M = V, Cr, Mo, W) as a function of W_{fill}. The dotted line shows the voltage change trend. W_{fill} of 2H-phase $V_{0.5}Cr_{1.5}S_4$, $VCrS_4$ and $V_{1.75}Cr_{0.25}S_4$ are much lower than that of Group-VIB 2H-MS_2.

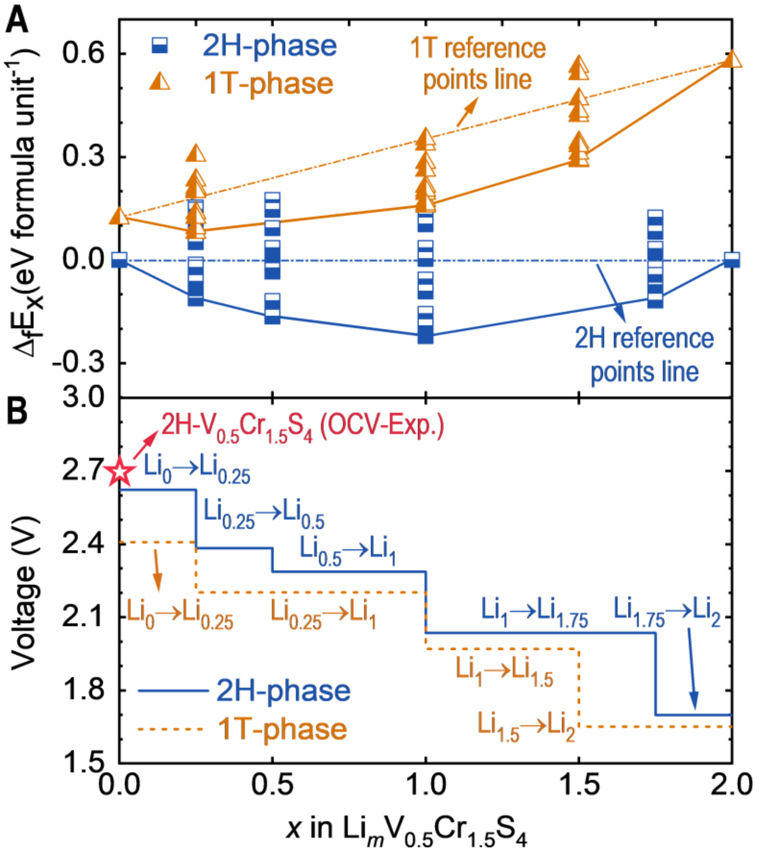

Figure S13. (A) Formation energies ($\Delta_f E_x$) and (B) voltages of 1T- and 2H-$V_{0.5}Cr_{1.5}S_4$ are shown as a function of Li-concentration ($Li_mV_{0.5}Cr_{1.5}S_4$). Yellow and blue solid lines indicate the constructed convex hull of the 1T-phase and 2H-phase, respectively. For each Li concentration point on the convex hull, we only list the lowest 10 configurational energies.

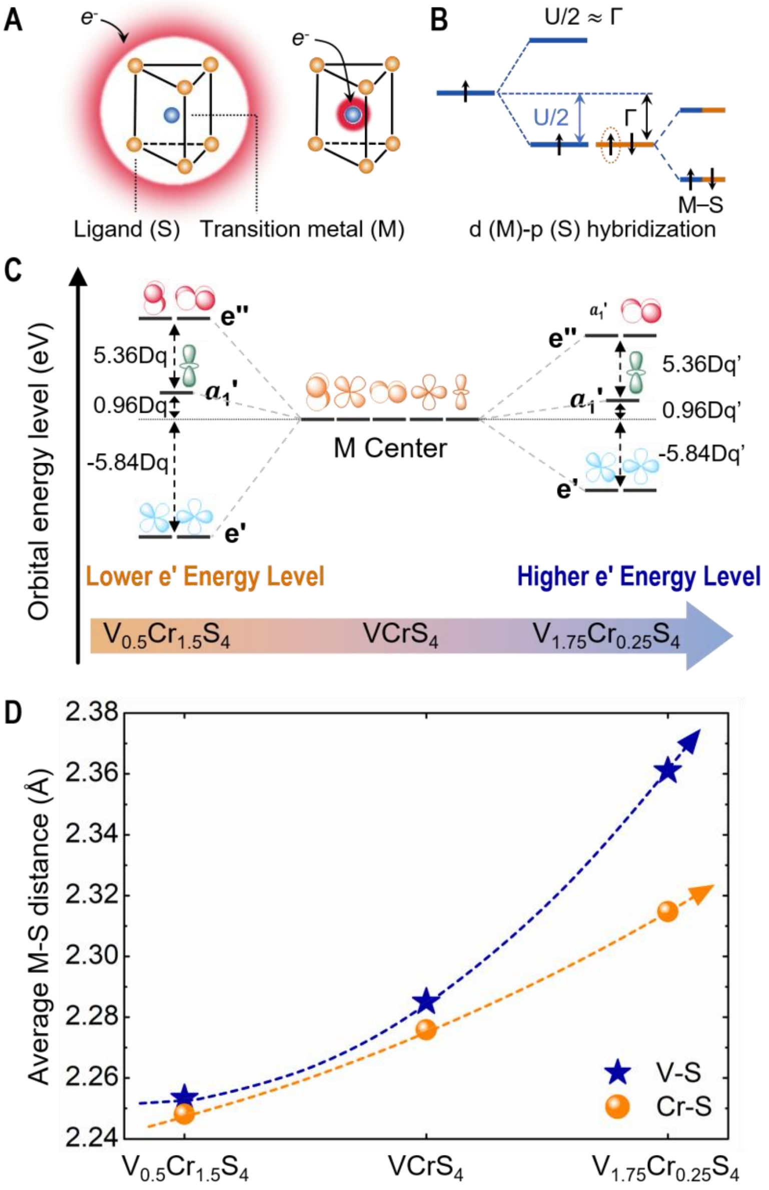

Figure S14. (A) Energy level of Cr/V e′ orbitals depending on the value of $-5.84 \times \frac{Z_L e^2 r_i^4}{6R^5}$. (B) Trends of average Cr/V-S distance in three components of 2H-$V_xCr_{2-x}S_4$ (x = 0.5, 1, 1.75). When moving from 2H-$V_{0.5}Cr_{1.5}S_4$ to 2H-$V_{1.75}Cr_{0.25}S_4$, the increase of average V-S distance is faster than that of Cr-S distance, resulting in the dominated participation of V-3d/S-2p during lithiation of 2H-$V_{1.75}Cr_{0.25}S_4$ cathode.

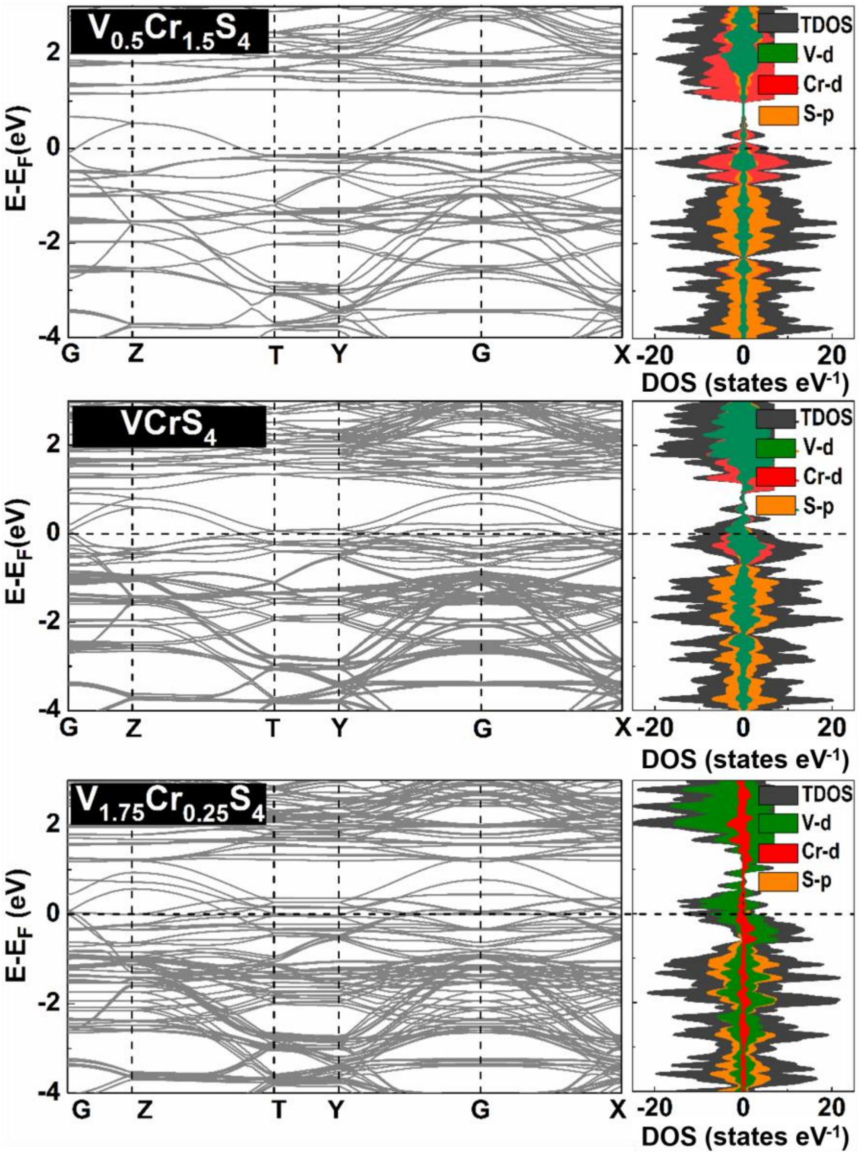

Figure S15. The band structures and partial density of states (PDOS) of 2H-$V_{0.5}Cr_{1.5}S_4$ (up), 2H-$VCrS_4$ (middle) and 2H-$V_{1.75}Cr_{0.25}S_4$ (down) systems.

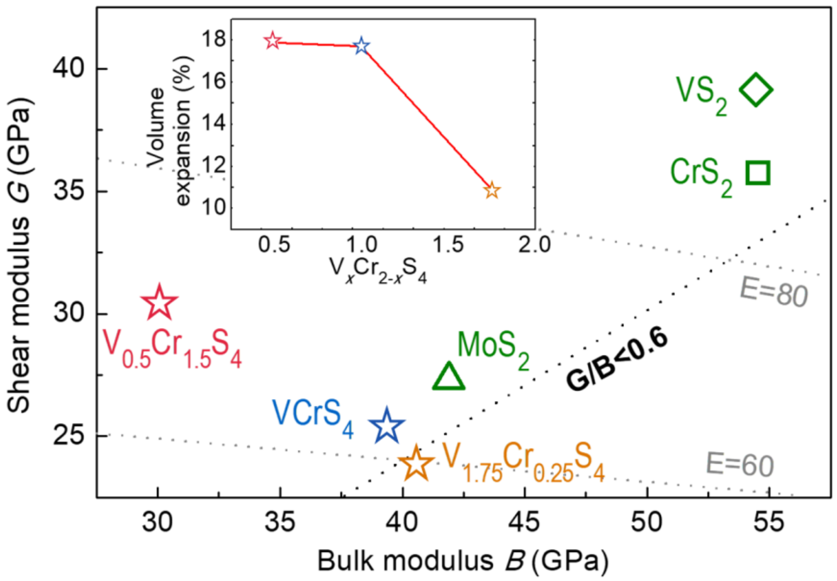

Figure S16. The shear modulus (*G*) as a function of bulk modulus (*B*) for 2H-$V_xCr_{2-x}S_4$. The dashed lines are the iso-Young's modulus lines in GPa. *G* and *B* values of conventional MS_2 (M = Mo, Cr, V) structures are also presented for comparison. The figure in the upper left corner shows the volume expansion ratio of the $Li_2V_xCr_{2-x}S_4$ discharge state. The lower *G*/*B* (~0.59) and higher *v* (~0.25) indicate that 2H-$V_{1.75}Cr_{0.25}S_4$ is less brittle, which is beneficial to the cycling stability.

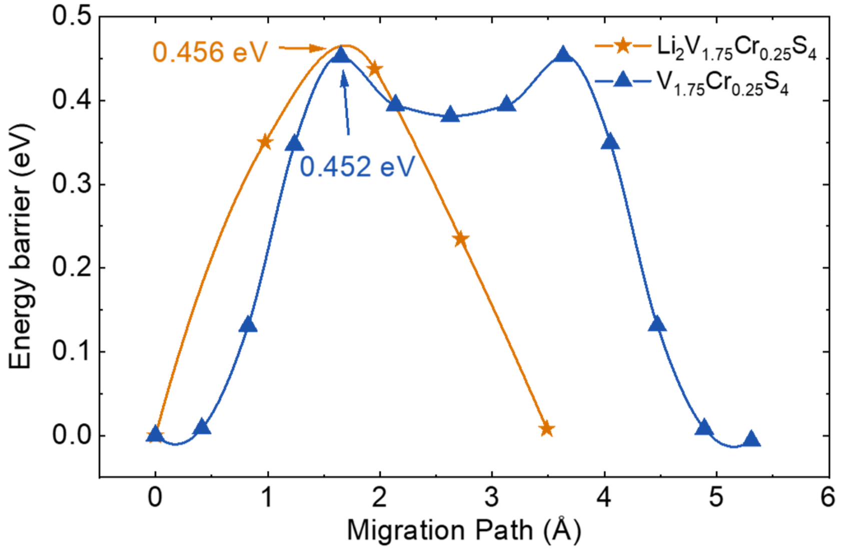

Figure S17. Li^+ diffusion barrier in the bulk of charged $V_{1.75}Cr_{0.25}S_4$ and discharged $Li_2V_{1.75}Cr_{0.25}S_4$ along the path of the hollow site to Cr/V top site is 0.452 eV and 0.456 eV, respectively. The calculated Li^+ diffusion barriers in both fully discharged $Li_2V_{1.75}Cr_{0.25}S_4$ and charged $V_{1.75}Cr_{0.25}S_4$ are close to those in common cathodes (< 0.6 eV) which have been widely used in battery applications.

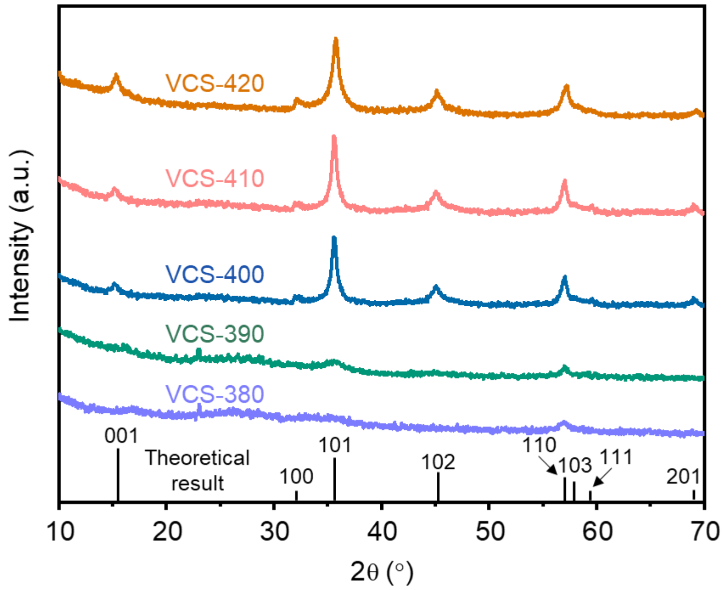

Figure S18. XRD patterns of VCS-380, VCS-390, VCS-400, VCS-410, and VCS-420 samples (named as VCS-temperature) with various hydrothermal synthesis temperatures, accompanied with relevant theoretical result. It is found that at 390 K or 380 K, not obvious peaks could be found for the synthetic products. This change tendency confirms that 400 K is the receptible temperature limit for successful hydrothermal preparation in this work.

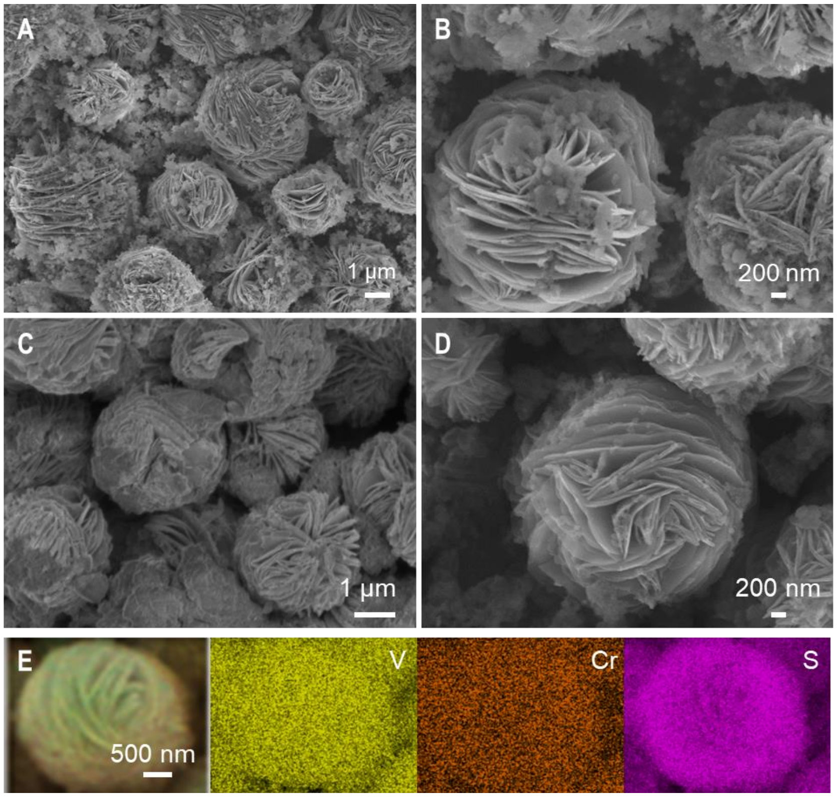

Figure S19. SEM images of (A to B) VCS-400 and (C to D) VCS-450, accompanied with (E) EDS mapping results of VCS-400.

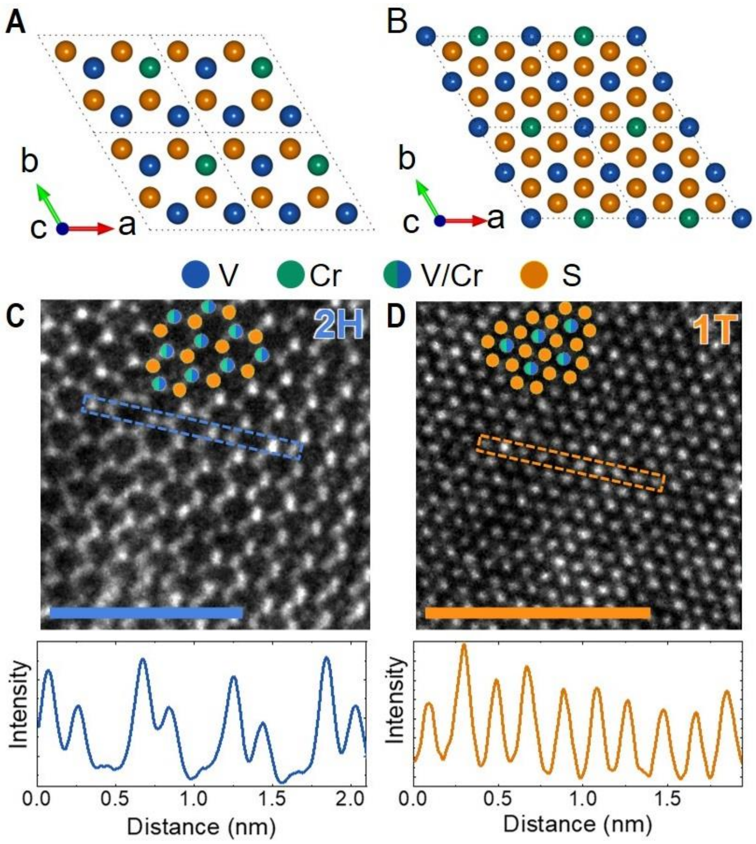

Figure S20. Schematic diagram of structures for (A) 2H-$V_{1.75}Cr_{0.25}S_4$ and (B) 1T-$V_{1.75}Cr_{0.25}S_4$ viewed from the [001] crystallographic direction. The TEM image and the corresponding line profile of (C) 2H-$V_{1.75}Cr_{0.25}S_4$ and (D) 1T-$V_{1.75}Cr_{0.25}S_4$. Scale bar, 2 nm.

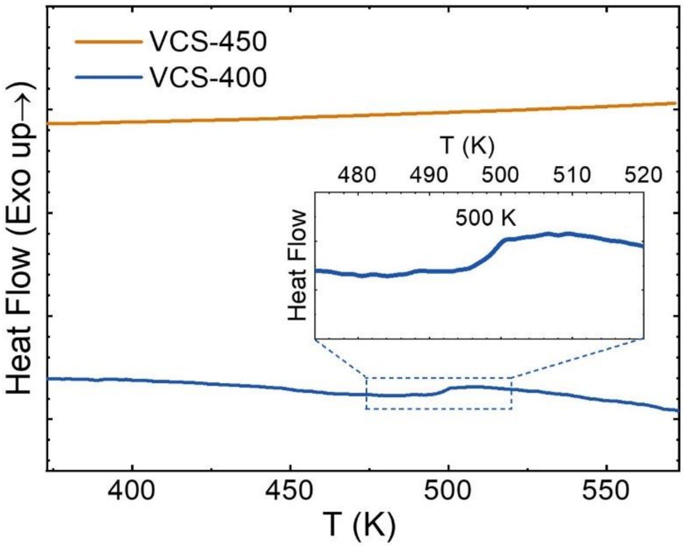

Figure S21. The DSC profiles of VCS-400 and VCS-450 in Ar atmosphere. The exothermic peak of VCS-400 corresponded to the 2H–1T phase transition behaviors of $V_{1.75}Cr_{0.25}S_4$, when the actual temperature above 500 K is quite close to the theoretical value.

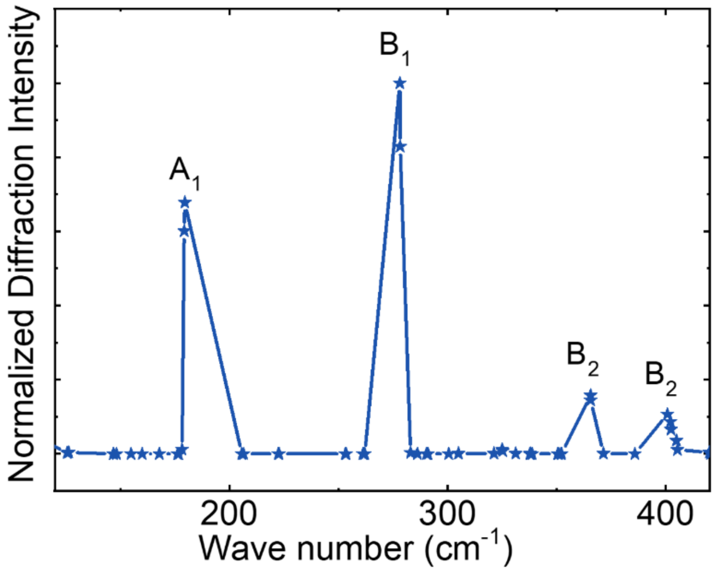

Figure S22. The calculated Raman spectrum of 2H-$V_{1.75}Cr_{0.25}S_4$ structure. The Raman active modes (A_1, B_1 and B_2) are shown separately. All spectra are normalized by the integrated intensity of maximum intensity mode to enable comparison of the relative intensity.

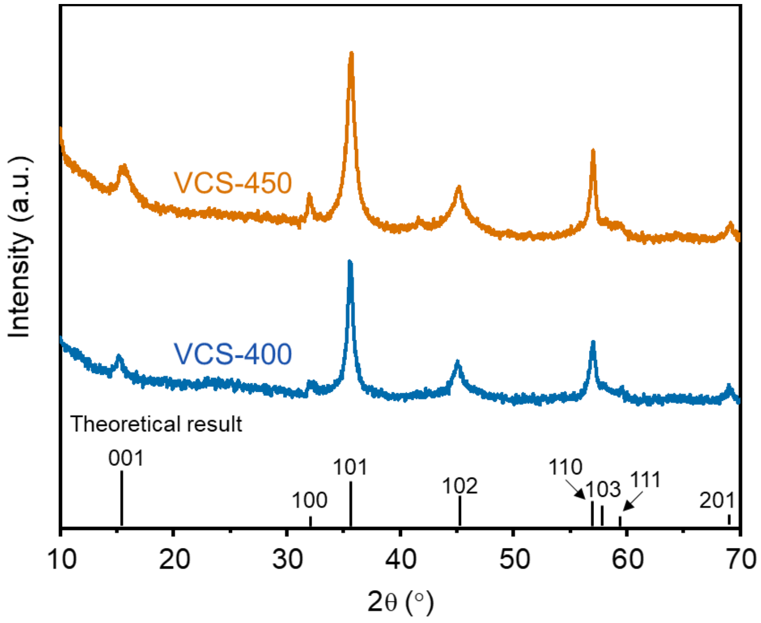

Figure S23. XRD patterns of VCS-450 and VCS-400 accompanied with relevant theoretical result. Both samples matched well with the calculated pattern of $P\overline{3}m1$ space group of 1T-phase, since the relatively low 2H-phase content in VCS-400 resulted in the absence of obvious characteristic XRD peaks.

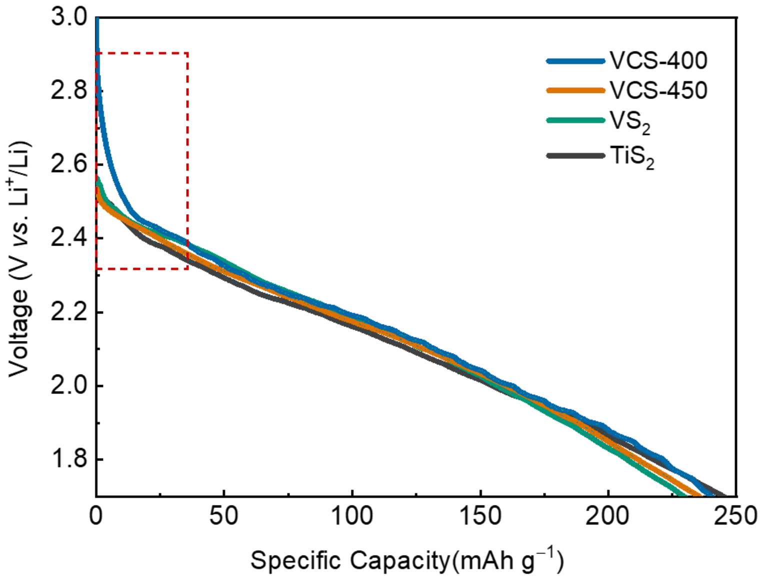

Figure S24. The initial discharge curves of Li-metal batteries based on VCS-400, VCS-450, 1T-VS_2, and commercial TiS_2 cathodes. During the initial discharge stage (red box region), the VCS-400 (1T+2H-mixed phase $V_{1.75}Cr_{0.25}S_4$) cell possessed higher voltage over VCS-450 (pure 1T-phase $V_{1.75}Cr_{0.25}S_4$) and 1T-phase VS_2, which evidenced the Li^+-intercalation voltage advantage of 2H-$V_{1.75}Cr_{0.25}S_4$ benefitted from the p-type alloying strategy.

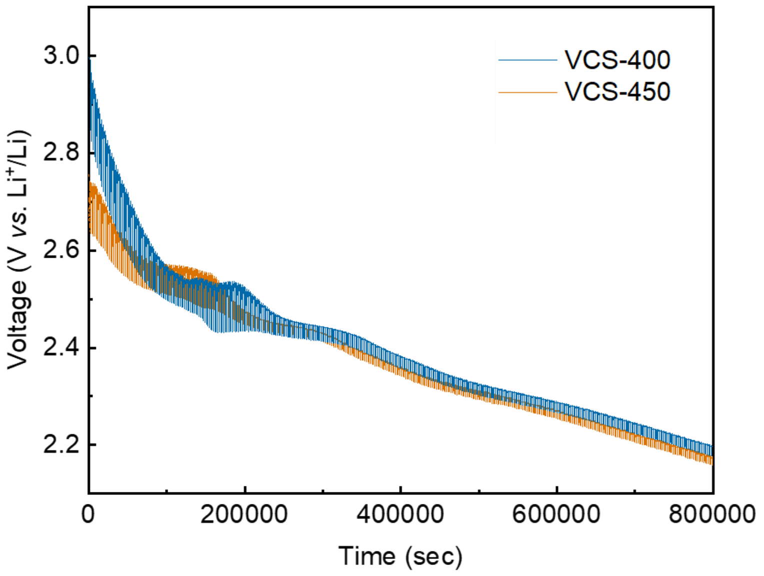

Figure S25. GITT curves of VCS-400 and VCS-450 cathodes during the initial discharge stage. It also evidences the higher Li^+-intercalation voltage of CVS-400, which should be attributed to the existing of 2H-$V_{1.75}Cr_{0.25}S_4$ content, albeit in a form of 1T/2H-mixed phase.

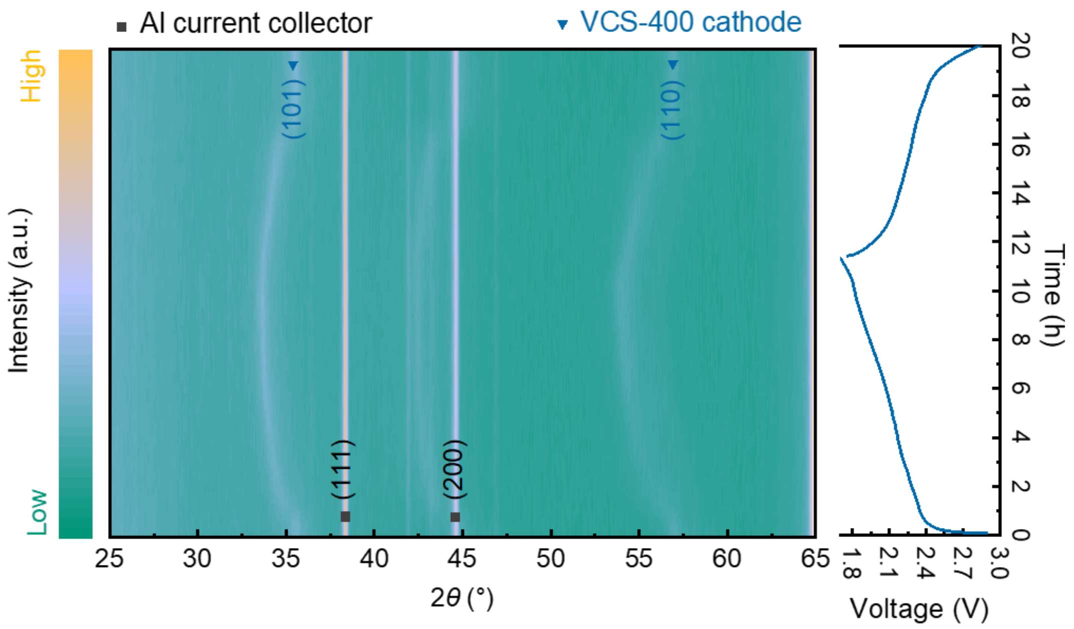

Figure S26. *In-situ* XRD patterns of VCS-400 cathodes during lithiation and delithiation processes. Compared with the initial state, the main diffraction peaks of VCS-400 at 35.7° and 57.0° were observed to slightly shift toward lower angles during the lithiation process (to 1.7 V). It agrees with its intercalation-type Li-storage mechanism, since the intercalation of Li^+ slightly enlarges the unit cell. Subsequently, during the delithiation process (to 2.8 V), the main diffraction peaks shifted toward higher angles until recovering to their initial state. The stable peak intensity indicates that no obvious conversion reaction occurs during the cycling process, confirming the high structural stability and reversibility of VCS-400. In addition, two strong peaks stabilized at 38.3° and 44.6°are assigned to the Al (JCPDS Card No. 04-0787) current collector.

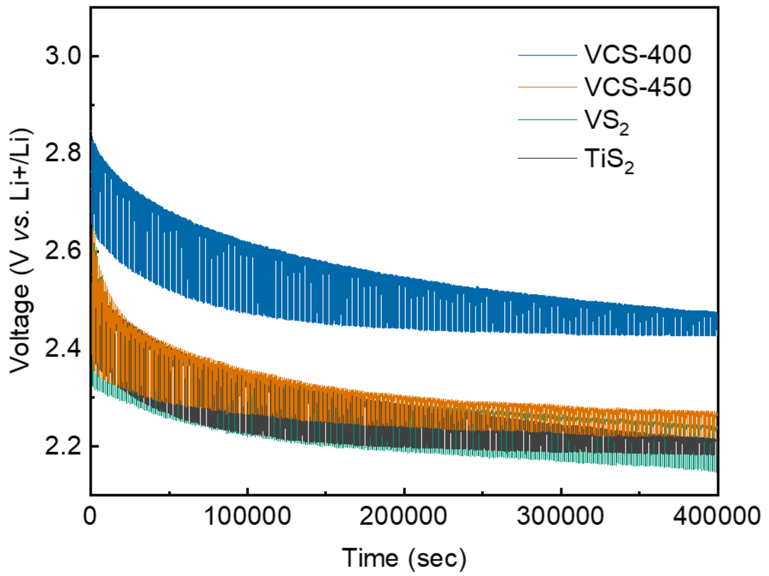

Figure S27. GITT curves of solid-state batteries based on VCS-400 (1T/2H-mixed $V_{1.75}Cr_{0.25}S_4$), VCS-450 (1T-$V_{1.75}Cr_{0.25}S_4$), 1T-VS_2, and commercial TiS_2 cathodes. We have fabricated solid-state batteries based on Li_3PS_4 (LPS) electrolytes using various MX_2 cathodes. The GITT results evidence the higher Li^+-intercalation voltage of VCS-400 than other cathodes. It should be attributed to the existing of 2H-$V_{1.75}Cr_{0.25}S_4$ content in VCS-400, which possesses voltage advantage over 1T-$V_{1.75}Cr_{0.25}S_4$ and 1T-VS_2, evidencing the effect of p-type alloying strategy for 2H rather than 1T phase.

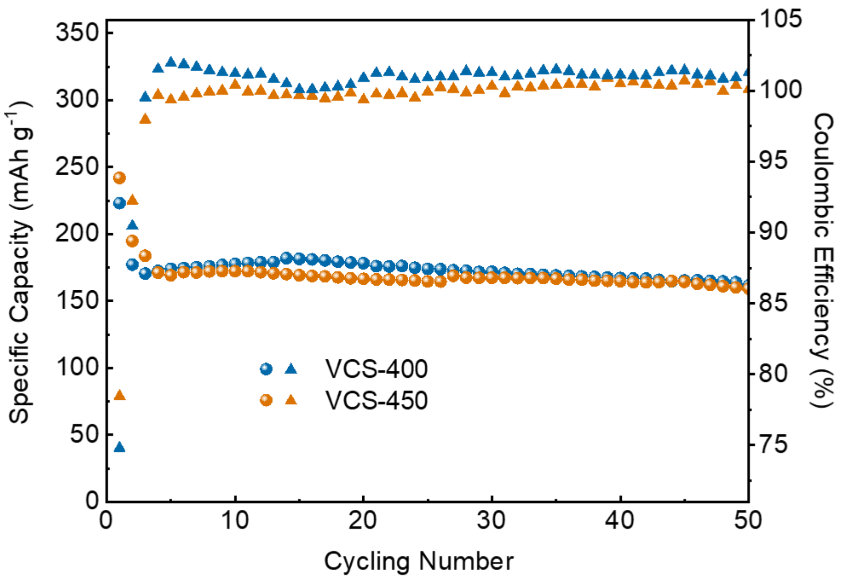

Figure S28. Discharge capacities and coulombic efficiencies (CE) of the VCS-400 and VCS-450 cathodes during cycles at room-temperature, with a 0.1 C rate and a 1.7–2.8 V voltage range.

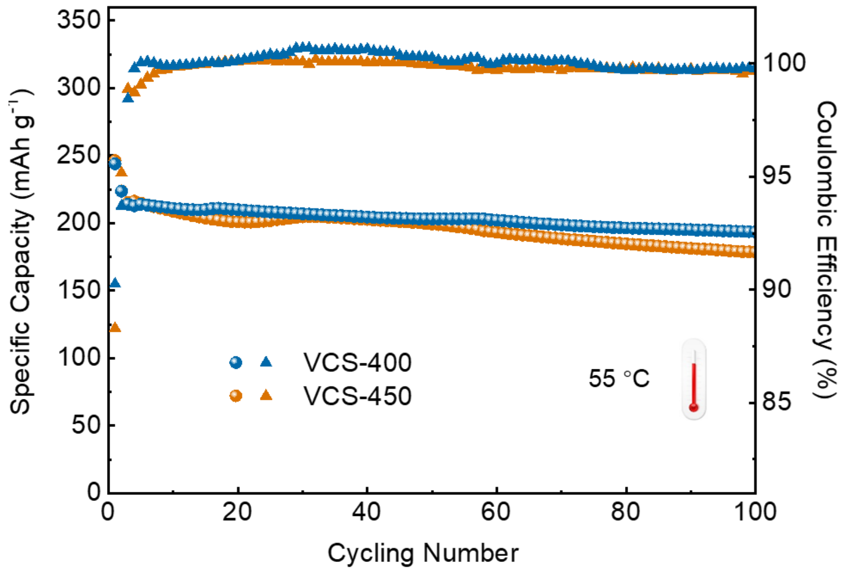

Figure S29. The discharge capacities and coulombic efficiencies (CE) of VCS-400 and VCS-450 during cycles at 55°C, with a 0.5 C rate and a 1.7–2.8 V voltage range. Both two cathodes delivered high initial discharge capacities and coulombic efficiencies at high temperature, demonstrating good cycling stability.

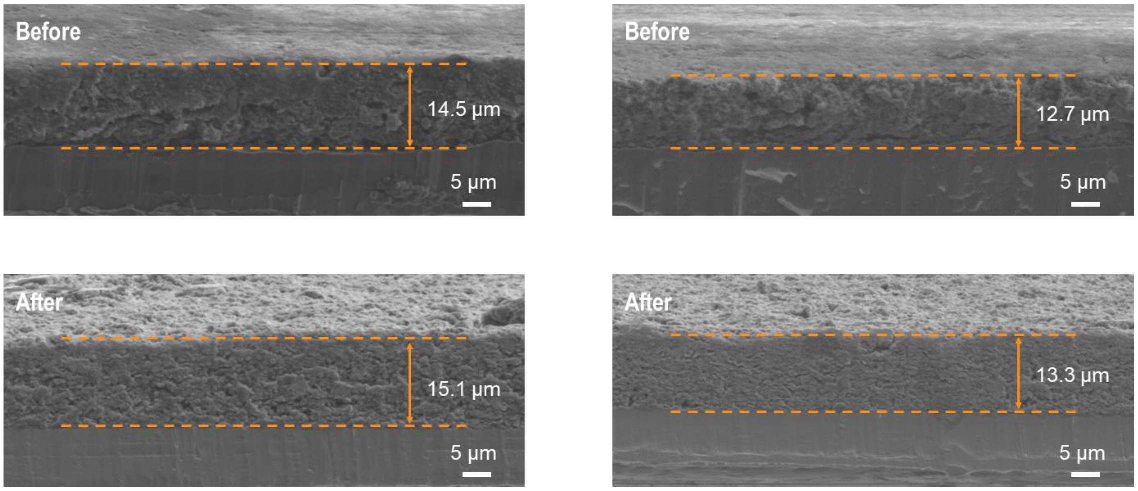

Figure S30. Cross-sectional SEM images of the (left) VCS-400 and (right) VCS-450 electrode pieces before and after discharging to 1.7 V.

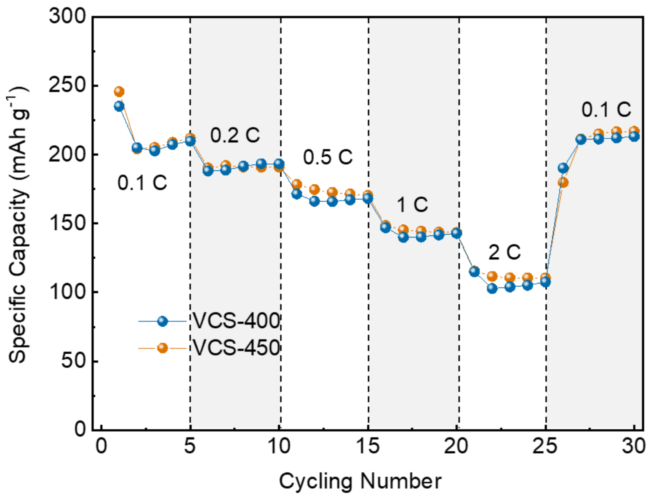

Figure S31. Rate capabilities of VCS-400 and VCS-450 cathodes. It is observed that VCS-400 demonstrates a similar rate performance as VCS-450. When the rate increased to 2 C, their discharge capacities maintained around 110 mAh g^{-1}, and their capacities recovered to more than 200 mAh g^{-1} as the rate returned to 0.1C. Obviously, the same intercalation-type Li-storage mechanism together with same theoretical specific capacities give rise to their similar rate capabilities.

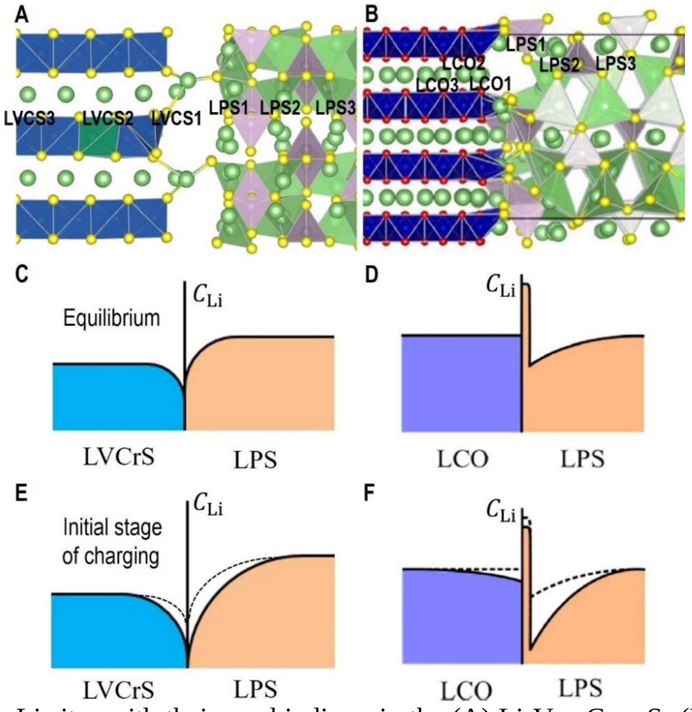

Figure S32. Possible Li sites with their used indices, in the (A) $Li_2V_{1.75}Cr_{0.25}S_4$ (LVCS) (001)/Li_3PS_4 (LPS) (010), (B) $LiCoO_2$ (LCO) (110)/LPS (010) [23] interface structures. Schematic illustrations of the interfacial Li^+ concentrations of the (C) LVCS (001)/LPS (010) and (D) LCO (110)/LPS (010) [23] at equilibrium. The interfacial Li^+ concentrations of the (E) LVCS (001)/LPS (010) and (F) LCO (110)/LPS (010) [23] at the initial charging stage, and the interfacial Li^+ concentrations at equilibrium are also shown here with dashed lines for comparison. C_{Li} indicate the Li^+ concentrations on the interfaces.

Supporting Tables

Table S1. Lattice constants (Å), average M–X bond-length (Å), and the energy difference between 1T and 2H phase (ΔE_{1T-2H}) of MX_2 (M = Cr, Mo, W, V, Nb, Ta; X = S, Se).

		Lattice constants (Å)			Average bond-length (Å)	ΔE_{1T-2H} (meV f.u.$^{-1}$)
		a	b	c		
VS_2	2H	3.158	3.158	5.983	2.351	40
	1T	3.174	3.174	5.835	2.347	
NbS_2	2H	3.336	3.336	6.091	2.483	36
	1T	3.348	3.348	5.817	2.483	
TaS_2	2H	3.321	3.321	6.139	2.475	51
	1T	3.348	3.348	5.917	2.473	
CrS_2	2H	3.019	3.019	6.044	2.280	533
	1T	3.040	3.040	5.997	2.293	
MoS_2	2H	3.169	3.169	6.172	2.408	815
	1T	3.183	3.183	5.917	2.421	
WS_2	2H	3.175	3.175	6.206	2.416	871
	1T	3.194	3.194	6.021	2.424	
VSe_2	2H	3.315	3.315	6.345	2.488	18
	1T	3.320	3.320	6.247	2.482	
$NbSe_2$	2H	3.460	3.460	6.393	2.612	56
	1T	3.447	3.447	6.244	2.611	
$TaSe_2$	2H	3.444	3.444	6.479	2.602	45
	1T	3.459	3.459	6.246	2.600	
$CrSe_2$	2H	3.181	3.181	6.370	2.418	418
	1T	3.198	3.198	6.363	2.431	
$MoSe_2$	2H	3.298	3.298	6.521	2.536	679
	1T	3.271	3.271	6.485	2.550	
WSe_2	2H	3.294	3.294	6.525	2.541	751
	1T	3.268	3.268	6.514	2.553	

Table S2. Absolute position of Fermi level (E_F, here work functions are calculated by aligning E_F relative to vacuum) and band gaps (E_g) of Group-VIB 2H-MX_2. Electrons integration (e^{int}) filled above valence band of 2H-MX_2 with one Li-2s electron is transferred into $M_{16}X_{32}$ ($Li_{0.0625}MX_2$).

	S			Se		
	E_F (eV)	E_g (eV)	e^{int} (e)	E_F (eV)	E_g (eV)	e^{int} (e)
Cr	-0.534	0.753	0.936	-0.516	0.722	0.887
Mo	-0.338	1.032	0.898	-0.305	0.979	0.888
W	-0.256	1.111	0.932	-0.227	1.086	0.910

Table S3. Splitting coefficient of atomic d orbitals in crystal fields of different symmetries.

Coordination number	Symmetry of site	d_{z^2}	$d_{x^2-y^2}$	d_{xy}	d_{xz}	d_{yz}
3	Triangular	−3.21Dq (a_1')	5.46Dq (e')	5.46Dq (e')	−3.86Dq (e'')	−3.86Dq (e'')
4	Tetrahedron	−2.67Dq (e)	−2.67Dq (e)	1.78Dq (t_2)	1.78Dq (t_2)	1.78Dq (t_2)
4	Plane square	−5.28Dq (a_{1g})	12.28Dq (b_{1g})	2.28Dq (b_{2g})	−4.64Dq (e_g)	−4.64Dq (e_g)
5	Trigonal bipyramid	7.07Dq (a_1')	−0.82Dq (e'^2)	−0.82Dq (e')	−2.72Dq (e'')	−2.72Dq (e'')
5	Square pyramid	0.86Dq (a_1)	9.14Dq (b_1)	−0.86Dq (b_2)	−4.57Dq (e)	−4.57Dq (e)
6	Octahedron	6Dq (e_g)	6Dq (e_g)	−4Dq (t_{2g})	−4Dq (t_{2g})	−4Dq (t_{2g})
6	Triangular prism	0.96Dq (a_1')	−5.84Dq (e')	−5.84Dq (e')	5.36Dq (e'')	5.36Dq (e'')
8	Cube	−5.34Dq (e_g)	−5.34Dq (e_g)	3.56Dq (t_{2g})	3.56Dq (t_{2g})	3.56Dq (t_{2g})

Expressed as fractions of the crystal field splitting parameter, Dq.

Table S4. Crystal field stabilization energies (CFSE) for octahedral complexes.

Number of d electron	Arrangement in weak ligand field		Arrangement in strong ligand field		CFSE	
	t_{2g}	e_g	t_{2g}	e_g	weak ligand field	strong ligand field
1	↑	−	↑	−	−4Dq	−4Dq
2	↑↑	−	↑↑	−	−8Dq	−8Dq
3	↑↑↑	−	↑↑↑	−	−12Dq	−12Dq
4	↑↑↑	↑	↑↓↑↑	−	−6Dq	−16Dq + P
5	↑↑↑	↑↑	↑↓↑↓↑	−	0	−20Dq + 2P
6	↑↓↑↑	↑↑	↑↓↑↓↑↓	↑	−4Dq	−24Dq + 2P
7	↑↓↑↓↑	↑↑	↑↓↑↓↑↓	↑	−8Dq	−18Dq + P
8	↑↓↑↓↑↓	↑↑	↑↓↑↓↑↓	↑↑	−12Dq	−12Dq
9	↑↓↑↓↑↓	↑↓↑	↑↓↑↓↑↓	↑↓↑	−6Dq	−6Dq
10	↑↓↑↓↑↓	↑↓↑↓	↑↓↑↓↑↓	↑↓↑↓	0	0

Expressed as fractions of the crystal field splitting parameter, Dq.

Table S5. Crystal field stabilization energies (CFSE) for trigonal prismatic complexes

Number of d electron	Arrangement in weak ligand field			Arrangement in strong ligand field			CFSE	
	e'	a_1'	e''	e'	a_1'	e''	weak ligand field	strong ligand field
1	↑	−	−	↑	−	−	−5.84Dq	−5.84Dq
2	↑↑	−	−	↑↑	−	−	−11.68Dq	−11.68Dq
3	↑↑	↑	−	↑↓↑	−	−	−10.72Dq	−17.52Dq + P
4	↑↓↑	↑	−	↑↓↑↓	−	−	−16.56Dq	−23.36Dq + P
5	↑↓↑↓	↑	−	↑↓↑↓	↑	−	−22.4Dq	−22.4Dq
6	↑↓↑↓	↑↓	−	↑↓↑↓	↑↓	−	−21.44Dq	−21.44Dq
7	↑↓↑↓	↑↓	↑	↑↓↑↓	↑↓	↑	−16.08Dq	−16.08Dq
8	↑↓↑↓	↑↓	↑↑	↑↓↑↓	↑↓	↑↑	−10.72Dq	−10.72Dq
9	↑↓↑↓	↑↓	↑↓↑	↑↓↑↓	↑↓	↑↓↑	−5.36Dq	−5.36Dq
10	↑↓↑↓	↑↓	↑↓↑↓	↑↓↑↓	↑↓	↑↓↑↓	0	0

Expressed as fractions of the crystal field splitting parameter, Dq.

Table S6. Calculated formation energies (E_f) and the energy above the convex hull (E_{above}) for the elements, the known compounds and the predicted $V_xCr_{2-x}S_4$ ($0 \le x \le 2$) in the V–Cr–S system.

Formula	Space group	E_f (eV f.u.$^{-1}$)	E_{above} (eV)
Cr_3S_4	$P2_1/m$	−1.03091	0
CrS_2	$P6_3/mmc$	−1.11744	0
V_3S	$P4_2/nbc$	−0.73258	0
V_3S_4	$P6_3/m$	−1.35646	0
V_5S_4	$I4/m$	−1.19976	0
VCr	$Cmmm$	−0.16102	0
VCr_3	$Fm\overline{3}m$	−0.12922	0
VS_2	$P6_3/mmc$	−1.26257	0
VS_4	$C2/c$	−1.03684	0
Cr	$Im\overline{3}m$	0	0
V	$Im\overline{3}m$	0	0
S	$P2/c$	0	0
$V_{0.5}Cr_{1.5}S_4$	$Cmcm$	−1.16135	0
$VCrS_4$	$Pmmn$	−1.19550	0
$V_{1.75}Cr_{0.25}S_4$	$Amm2$	−1.24344	0

Table S7. Calculated initial Li^+-intercalation voltage (V), average Li^+-intercalation voltage (V), theoretical specific capacity (mAh g^{-1}) and energy density (Wh kg^{-1}) of Li-free (charged) 2H-$V_xCr_{2-x}S_4$ ($0 \le x \le 2$) at electrode level, respectively. The specific charges are based on a reversible range (*m*) of Li^+ content per unit formula during charging/discharging. For comparison, the reported operation voltage (V, *vs.* Li/Li^+), theoretical specific capacity (mAh g^{-1}) and energy density (Wh kg^{-1}) of TiS_2 cathode is also presented [29, 42].

	$V_{0.5}Cr_{1.5}S_4$	$VCrS_4$	$V_{1.75}Cr_{0.25}S_4$	TiS_2 [29, 42]
Initial Li^+-intercalation voltage ($Li_{0.125}V_xCr_{2-x}S_4$)	2.623	2.702	2.767	~2.45
Average Li^+-intercalation voltage ($Li_2V_xCr_{2-x}S_4$)	2.173	2.241	2.382	~2.1
Reversible range (*m*)	2	2	2	1
Theoretical specific capacity	231.3	231.8	232.7	239.3
Energy density	502.6	519.5	554.3	502.5

Table S8. The calculated space group (SG), full elastic tensor (C_{ij}), bulk modulus (*B*), shear modulus (*G*), Young's modulus(*E*), Poisson's ratio (*v*) and Pugh's ratio (*G*/*B*) of $V_xCr_{2-x}S_4$ (x = 0.5, 1, 1.75) using the PBE functional, respectively.

Formula	$V_{0.5}Cr_{1.5}S_4$	$VCrS_4$	$V_{1.75}Cr_{0.25}S_4$
SG	*Cmcm*	*Pmmn*	*Amm*2
C_{ij} (GPa)	$\begin{bmatrix} 188.49 & 38.75 & -4.71 & 0 & 0 & 0 \\ 38.75 & 181.02 & -6.79 & 0 & 0 & 0 \\ -4.71 & -6.79 & 14.61 & 0 & 0 & 0 \\ 0 & 0 & 0 & 71.90 & 0 & 0 \\ 0 & 0 & 0 & 0 & 12.10 & 0 \\ 0 & 0 & 0 & 0 & 0 & 12.39 \end{bmatrix}$	$\begin{bmatrix} 21.06 & 17.86 & 20.59 & 0 & 0 & 0 \\ 17.86 & 168.68 & 38.23 & 0 & 0 & 0 \\ 20.59 & 38.23 & 176.10 & 0 & 0 & 0 \\ 0 & 0 & 0 & 9.94 & 0 & 0 \\ 0 & 0 & 0 & 0 & 66.40 & 0 \\ 0 & 0 & 0 & 0 & 0 & 8.08 \end{bmatrix}$	$\begin{bmatrix} 28.92 & 13.17 & 13.19 & 0 & 0 & 0 \\ 13.17 & 163.35 & 43.42 & 0 & 0 & 0 \\ 13.19 & 43.42 & 159.06 & 0 & 0 & 0 \\ 0 & 0 & 0 & 7.42 & 0 & 0 \\ 0 & 0 & 0 & 0 & 59.94 & 0 \\ 0 & 0 & 0 & 0 & 0 & 7.48 \end{bmatrix}$
B (GPa)	30.16	39.35	40.55
G (GPa)	30.39	25.27	23.80
E_Y (GPa)	68.25	62.44	59.73
v	0.12	0.24	0.25
G/*B*	1.01	0.64	0.59

Satisfy the criteria (*Cmcm*, *Pmmn* and *Amm*2): C_{11}>0, C_{22}>0, C_{33}>0, C_{44}>0, C_{55}>0, C_{66}>0, [C_{11}+C_{22}+C_{33}+2(C_{12}+C_{13}+C_{23})]>0, (C_{11}+C_{22}-2C_{12})>0, (C_{11}+C_{33}-2C_{13}) > 0, (C_{22}+C_{33}-2C_{23})>0. $V_xCr_{2-x}S_4$ (x = 0.5, 1 and 1.75) satisfy these criteria.

Table S9. Previous works on solvothermal preparation of VS_2 and relevant reaction conditions.

Literatures	Product	Method	Reaction temperature
J. Am. Chem. Soc. 2011, **133**, 17832	Nanosheets	Hydrothermal	433 K
J. Am. Chem. Soc. 2013, **135**, 8720	Flower-Like Nanosheets	Hydrothermal	433 K
Nano Energy 2015, **18**, 20	Nanosheets	Hydrothermal	433 K
Chem. Mater. 2016, **28**, 5587	Nanoplate Arrays	Hydrothermal	433 K
Nano Energy 2017, **35**, 396	Stacked Nanosheets	Hydrothermal	453 K
Adv. Mater. 2017, **29**, 1702061	Nanosheet Assemblies	Solvothermal	473 K
Adv. Energy Mater. 2017, **7**, 1601920	Nanosheets	Hydrothermal	453 K
Small 2018, **14**, 1703098	Nanosheets	Solvothermal	433 K
Energy Storage Mater. 2018, **11**, 1	Flower-Like Nanosheets	Hydrothermal	433 K
Energy Storage Mater. 2018, **12**, 61	Flower-Like Nanosheets	Hydrothermal	453 K
J. Mater. Chem. A 2019, **7**, 16330	Flower-Like Nanosheets	Hydrothermal	453 K
Small 2019, **15**, 1903904	Flower-Like Structure	Solvothermal	473 K
ACS Nano 2020, **14**, 5600	Nanosheets	Hydrothermal	433 K
J. Mater. Chem. A 2020, **8**, 9068	Arrays	Hydrothermal	433 K
J. Energy Chem. 2020, **42**, 34	Bio-Templated	Hydrothermal	433 K
Adv. Funct. Mater. 2021, **31**, 2008743	Flower-Like Nanosheets	Solvothermal	453 K
Appl. Surf. Sci. 2021, **544**, 148882	Nanosheets	Hydrothermal	453 K

Table S10. Calculated Li-vacancy formation energies E_v (eV) for $LVCS_n$, LCO_n, LPS_n, *n* represent the possible Li sites in $Li_2V_{1.75}Cr_{0.25}S_4$ (LVCS), $LiCoO_2$ (LCO) or Li_3PS_4 (LPS) structures. E_v of LVCS(001)/LPS(010) changes gently with a downward trend in the interface region, providing smooth Li^+ migration paths free from possible bottlenecks and reducing the interfacial resistance.

	LVCS(001)/LPS(010)							
Li site indices	LVCS-Bulk	LVCS3	LVCS2	LVCS1	LPS1	LPS2	LPS3	LPS-Bulk
E_v	1.89	1.81	1.74	1.53	1.37	2.16	2.80	3.20
	LCO(110)/LPS(010) [23]							
Li site indices	LCO-Bulk	LCO3	LCO2	LCO1	LPS1	LPS2	LPS3	LPS-Bulk
E_v	3.58	3.49	3.98	3.18	3.27	1.44	3.03	3.20

Appendixes: Optimized geometrical coordinates (POSCAR) of 2H-$V_xCr_{2-x}S_4$ (x = 0.5, 1, 1.75) and $Li_2V_{1.75}Cr_{0.25}S_2/Li_3PS_4$ structures

2H-$V_{0.5}Cr_{1.5}S_4$ (POSCAR)

```
1.0
   6.1270999908    0.0000000000    0.0000000000
   0.0000000000   10.6127996445    0.0000000000
   0.0000000000    0.0000000000   11.8843002319
   V   Cr    S
   4   12   32
Direct
     0.000000000     0.083379999     0.250000000
     0.000000000     0.916620016     0.750000000
     0.500000000     0.583379984     0.250000000
     0.500000000     0.416620016     0.750000000
     0.000000000     0.584540009     0.250000000
     0.000000000     0.415459991     0.750000000
     0.500000000     0.084540009     0.250000000
     0.500000000     0.915459991     0.750000000
     0.248490006     0.167109996     0.750000000
     0.751510024     0.832890034     0.250000000
     0.751510024     0.167109996     0.750000000
     0.248490006     0.832890034     0.250000000
     0.748489976     0.667109966     0.750000000
     0.251509994     0.332890004     0.250000000
     0.251509994     0.667109966     0.750000000
     0.748489976     0.332890004     0.250000000
     0.252629995     0.167649999     0.373569965
     0.747370005     0.832350016     0.626430035
     0.747370005     0.832350016     0.873569965
     0.252629995     0.167649999     0.126430035
     0.747370005     0.167649999     0.126430035
     0.252629995     0.832350016     0.873569965
     0.252629995     0.832350016     0.626430035
     0.747370005     0.167649999     0.373569965
     0.752629995     0.667649984     0.373569965
     0.247370005     0.332350016     0.626430035
     0.247370005     0.332350016     0.873569965
     0.752629995     0.667649984     0.126430035
     0.247370005     0.667649984     0.126430035
     0.752629995     0.332350016     0.873569965
     0.752629995     0.332350016     0.626430035
     0.247370005     0.667649984     0.373569965
     0.000000000     0.914950013     0.373579979
     0.000000000     0.085050002     0.626420021
     0.000000000     0.085050002     0.873579979
     0.000000000     0.914950013     0.126420021
     0.500000000     0.414950013     0.373579979
     0.500000000     0.585049987     0.626420021
     0.500000000     0.585049987     0.873579979
     0.500000000     0.414950013     0.126420021
     0.000000000     0.416779995     0.371140003
     0.000000000     0.583220005     0.628859997
     0.000000000     0.583220005     0.871140003
     0.000000000     0.416779995     0.128859997
     0.500000000     0.916779995     0.371140003
     0.500000000     0.083220005     0.628859997
     0.500000000     0.083220005     0.871140003
     0.500000000     0.916779995     0.128859997
```

2H-$VCrS_4$ (POSCAR)

```
1.0
11.7597883159223553  0.0000000000000000  0.0000000000000000
  0.0000000000000000  3.1011211419225670  0.0000000000000000
  0.0000000000000000  0.0000000000000000  5.3904936020108858
   V    Cr    S
   2     2     8
Direct
  0.5000000000000000  0.5000000000000000  0.4207726442495172
  0.0000000000000000  0.0000000000000000  0.5792273557504828
  0.0000000000000000  0.5000000000000000  0.0853779461341233
  0.5000000000000000  0.0000000000000000  0.9146220398658755
  0.8746681894502402  0.5000000000000000  0.4119336790180697
  0.1253318105497527  0.5000000000000000  0.4119336790180697
  0.6253318105497598  0.0000000000000000  0.5880663209819303
  0.3746681894502402  0.0000000000000000  0.5880663209819303
  0.8772103945506728  0.0000000000000000  0.9197467018511105
  0.1227896054493272  0.0000000000000000  0.9197467018511105
  0.6227896054493272  0.5000000000000000  0.0802532981488895
  0.3772103945506800  0.5000000000000000  0.0802532981488895
```

2H-$V_{1.75}Cr_{0.25}S_4$ (POSCAR)

```
1.0
 11.9403526921167700  0.0000000000000000   0.0000000000000000
 0.0000000000000000  6.3002569767059109   0.0000000000000000
 0.0000000000000000  0.0000000000000000  10.9167728595302496
    V    Cr    S
    14    2    32
Direct
-0.0000000000000000  0.7548906407486000  0.1149529462312310
 -0.0000000000000000  0.2451093592514003  0.1149529462312310
 -0.0000000000000000  0.2548906407485996  0.6149529462312310
 -0.0000000000000000  0.7451093592514000  0.6149529462312310
  0.5000000000000000  0.7500419287902355  0.2834280954850423
  0.5000000000000000  0.2499580712097647  0.2834280954850423
  0.5000000000000000  0.2500419287902351  0.7834280954850423
  0.5000000000000000  0.7499580712097645  0.7834280954850423
  0.5000000000000000  -0.0000000000000000  0.0334144388150265
  0.5000000000000000  0.5000000000000000  0.5334144688150217
  0.5000000000000000  0.5000000000000000  0.0333876237249829
  0.5000000000000000  -0.0000000000000000  0.5333876007249775
 -0.0000000000000000  -0.0000000000000000  0.8697859937861533
 -0.0000000000000000  0.5000000000000000  0.3697859937861532
 -0.0000000000000000  0.5000000000000000  0.8665776811676987
 -0.0000000000000000  -0.0000000000000000  0.3665776811676991
  0.8782671824285830  0.7514050160872937  0.2836936110182006
  0.1217328175714171  0.2485949839127065  0.2836936110182006
  0.8782671824285830  0.2485949839127065  0.2836936110182006
  0.1217328175714171  0.7514050160872937  0.2836936110182006
  0.8782671824285830  0.2514050160872937  0.7836936110182011
  0.1217328175714171  0.7485949839127063  0.7836936110182011
  0.8782671824285830  0.7485949839127063  0.7836936110182011
  0.1217328175714171  0.2514050160872937  0.7836936110182011
  0.6245840240300001  0.7499417273565024  0.1168076292670565
  0.3754159759699999  0.2500582726434974  0.1168076292670565
  0.6245840240300001  0.2500582726434974  0.1168076292670565
  0.3754159759699999  0.7499417273565024  0.1168076292670565
  0.6245840240300001  0.2499417273565026  0.6168076292670562
  0.3754159759699999  0.7500582726434976  0.6168076292670562
  0.6245840240300001  0.7500582726434976  0.6168076292670562
  0.3754159759699999  0.2499417273565026  0.6168076292670562
  0.8741366950404744  -0.0000000000000000  0.0332311789051236
  0.1258633049595254  -0.0000000000000000  0.0332311789051236
  0.8741366950404744  0.5000000000000000  0.5332312059051292
  0.1258633049595254  0.5000000000000000  0.5332312059051292
  0.6245844472426949  -0.0000000000000000  0.8667508038303927
  0.3754155527573048  -0.0000000000000000  0.8667508038303927
  0.6245844472426949  0.5000000000000000  0.3667508038303925
  0.3754155527573048  0.5000000000000000  0.3667508038303925
  0.8782688823400877  0.5000000000000000  0.0322688918581004
  0.1217311176599123  0.5000000000000000  0.0322688918581004
  0.8782688823400877  -0.0000000000000000  0.5322688808581032
  0.1217311176599123  -0.0000000000000000  0.5322688808581032
  0.6246126591580876  0.5000000000000000  0.8667877293726646
  0.3753873408419125  0.5000000000000000  0.8667877293726646
  0.6246126591580876  -0.0000000000000000  0.3667877293726647
  0.3753873408419125  -0.0000000000000000  0.3667877293726647
```

$Li_2V_{1.75}Cr_{0.25}S_2/Li_3PS_4$ interface structure (POSCAR)

1.0

12.4654998779	0.0000000000	0.0000000000
0.0000000000	12.9299001694	0.0000000000
0.0000000000	0.0000000000	67.5526962280

Li	V	Cr	S	P
108	42	6	176	20

Direct

0.249899998	0.000000000	0.332549989
0.249899998	0.500000000	0.332549989
0.249149993	0.250000000	0.333000004
0.249149993	0.750000000	0.333000004
0.750100017	0.000000000	0.332549989
0.750100017	0.500000000	0.332549989
0.750850022	0.250000000	0.333000004
0.750850022	0.750000000	0.333000004
0.250440001	0.125190005	0.373910010
0.250440001	0.625190020	0.373910010
0.250440001	0.374810010	0.373910010
0.250440001	0.874809980	0.373910010
0.749559999	0.125190005	0.373910010
0.749559999	0.625190020	0.373910010
0.749559999	0.374810010	0.373910010
0.749559999	0.874809980	0.373910010
0.249149993	0.000000000	0.250209987
0.249149993	0.000000000	0.415789992
0.249149993	0.500000000	0.250209987
0.249149993	0.500000000	0.415789992
0.249899998	0.250000000	0.249760002
0.249899998	0.250000000	0.415329993
0.249899998	0.750000000	0.249760002
0.249899998	0.750000000	0.415329993
0.750850022	0.000000000	0.250209987
0.750850022	0.000000000	0.415789992
0.750850022	0.500000000	0.250209987
0.750850022	0.500000000	0.415789992
0.750100017	0.250000000	0.249760002
0.750100017	0.250000000	0.415329993
0.750100017	0.750000000	0.249760002
0.750100017	0.750000000	0.415329993
0.250440001	0.124810003	0.291119993
0.250440001	0.124810003	0.456699997
0.250440001	0.624809980	0.291119993
0.250440001	0.624809980	0.456699997
0.250440001	0.375189990	0.291119993
0.250440001	0.375189990	0.456699997
0.250440001	0.875190020	0.291119993
0.250440001	0.875190020	0.456699997
0.749559999	0.124810003	0.291119993
0.749559999	0.124810003	0.456699997
0.749559999	0.624809980	0.291119993
0.749559999	0.624809980	0.456699997
0.749559999	0.375189990	0.291119993
0.749559999	0.375189990	0.456699997
0.749559999	0.875190020	0.291119993
0.749559999	0.875190020	0.456699997
0.190510005	0.331250012	0.518270016
0.690509975	0.331250012	0.518270016
0.190510005	0.331250012	0.635389984
0.690509975	0.331250012	0.635389984

0.190510005	0.331250012	0.752510011
0.690509975	0.331250012	0.752510011
0.309489995	0.668749988	0.510580003
0.809490025	0.668749988	0.510580003
0.309489995	0.668749988	0.627709985
0.809490025	0.668749988	0.627709985
0.309489995	0.668749988	0.744830012
0.809490025	0.668749988	0.744830012
0.440510005	0.168750003	0.510580003
0.940509975	0.168750003	0.510580003
0.440510005	0.168750003	0.627709985
0.940509975	0.168750003	0.627709985
0.440510005	0.168750003	0.744830012
0.940509975	0.168750003	0.744830012
0.059489999	0.831250012	0.518270016
0.559490025	0.831250012	0.518270016
0.059489999	0.831250012	0.635389984
0.559490025	0.831250012	0.635389984
0.059489999	0.831250012	0.752510011
0.559490025	0.831250012	0.752510011
0.309489995	0.668749988	0.576830029
0.809490025	0.668749988	0.576830029
0.309489995	0.668749988	0.693949997
0.809490025	0.668749988	0.693949997
0.190510005	0.331250012	0.569149971
0.690509975	0.331250012	0.569149971
0.190510005	0.331250012	0.686269999
0.690509975	0.331250012	0.686269999
0.059489999	0.831250012	0.569149971
0.559490025	0.831250012	0.569149971
0.059489999	0.831250012	0.686269999
0.559490025	0.831250012	0.686269999
0.440510005	0.168750003	0.576830029
0.940509975	0.168750003	0.576830029
0.440510005	0.168750003	0.693949997
0.940509975	0.168750003	0.693949997
0.250000000	0.000000000	0.514429986
0.750000000	0.000000000	0.514429986
0.250000000	0.000000000	0.631550014
0.750000000	0.000000000	0.631550014
0.250000000	0.000000000	0.748669982
0.750000000	0.000000000	0.748669982
0.000000000	0.500000000	0.514429986
0.500000000	0.500000000	0.514429986
0.000000000	0.500000000	0.631550014
0.500000000	0.500000000	0.631550014
0.000000000	0.500000000	0.748669982
0.500000000	0.500000000	0.748669982
0.250000000	0.000000000	0.572990000
0.750000000	0.000000000	0.572990000
0.250000000	0.000000000	0.690110028
0.750000000	0.000000000	0.690110028
0.000000000	0.500000000	0.572990000
0.500000000	0.500000000	0.572990000
0.000000000	0.500000000	0.690110028
0.500000000	0.500000000	0.690110028
0.000000000	0.374769986	0.318419993
0.000000000	0.874769986	0.318419993
0.000000000	0.125229999	0.318419993
0.000000000	0.625230014	0.318419993

0.000000000	0.124770001	0.235640004
0.000000000	0.124770001	0.401210010
0.000000000	0.624769986	0.235640004
0.000000000	0.624769986	0.401210010
0.000000000	0.375230014	0.235640004
0.000000000	0.375230014	0.401210010
0.000000000	0.875230014	0.235640004
0.000000000	0.875230014	0.401210010
0.500000000	0.375039995	0.346439987
0.500000000	0.875039995	0.346439987
0.500000000	0.124959998	0.346439987
0.500000000	0.624960005	0.346439987
0.500000000	0.125039995	0.263650000
0.500000000	0.125039995	0.429230005
0.500000000	0.625039995	0.263650000
0.500000000	0.625039995	0.429230005
0.500000000	0.374960005	0.263650000
0.500000000	0.374960005	0.429230005
0.500000000	0.874960005	0.263650000
0.500000000	0.874960005	0.429230005
0.500000000	0.000000000	0.305099994
0.500000000	0.500000000	0.305099994
0.500000000	0.250000000	0.222310007
0.500000000	0.250000000	0.387890011
0.500000000	0.750000000	0.222310007
0.500000000	0.750000000	0.387890011
0.500000000	0.250000000	0.305059999
0.500000000	0.750000000	0.305059999
0.500000000	0.000000000	0.222269997
0.500000000	0.000000000	0.387849987
0.500000000	0.500000000	0.222269997
0.500000000	0.500000000	0.387849987
0.000000000	0.000000000	0.277469993
0.000000000	0.000000000	0.443040013
0.000000000	0.500000000	0.277469993
0.000000000	0.500000000	0.443040013
0.000000000	0.250000000	0.360249996
0.000000000	0.750000000	0.360249996
0.000000000	0.250000000	0.278340012
0.000000000	0.250000000	0.443910003
0.000000000	0.750000000	0.278340012
0.000000000	0.750000000	0.443910003
0.000000000	0.000000000	0.361119986
0.000000000	0.500000000	0.361119986
0.884469986	0.377209991	0.346579999
0.884469986	0.877210021	0.346579999
0.115529999	0.122790001	0.346579999
0.115529999	0.622789979	0.346579999
0.884469986	0.122790001	0.346579999
0.884469986	0.622789979	0.346579999
0.115529999	0.377209991	0.346579999
0.115529999	0.877210021	0.346579999
0.884469986	0.127210006	0.263790011
0.884469986	0.127210006	0.429360002
0.884469986	0.627210021	0.263790011
0.884469986	0.627210021	0.429360002
0.115529999	0.372790009	0.263790011
0.115529999	0.372790009	0.429360002
0.115529999	0.872789979	0.263790011
0.115529999	0.872789979	0.429360002

0.884469986	0.372790009	0.263790011
0.884469986	0.372790009	0.429360002
0.884469986	0.872789979	0.263790011
0.884469986	0.872789979	0.429360002
0.115529999	0.127210006	0.263790011
0.115529999	0.127210006	0.429360002
0.115529999	0.627210021	0.263790011
0.115529999	0.627210021	0.429360002
0.617709994	0.374859989	0.318870008
0.617709994	0.874859989	0.318870008
0.382290006	0.125139996	0.318870008
0.382290006	0.625140011	0.318870008
0.617709994	0.125139996	0.318870008
0.617709994	0.625140011	0.318870008
0.382290006	0.374859989	0.318870008
0.382290006	0.874859989	0.318870008
0.617709994	0.124860004	0.236080006
0.617709994	0.124860004	0.401650012
0.617709994	0.624859989	0.236080006
0.617709994	0.624859989	0.401650012
0.382290006	0.375140011	0.236080006
0.382290006	0.375140011	0.401650012
0.382290006	0.875140011	0.236080006
0.382290006	0.875140011	0.401650012
0.617709994	0.375140011	0.236080006
0.617709994	0.375140011	0.401650012
0.617709994	0.875140011	0.236080006
0.617709994	0.875140011	0.401650012
0.382290006	0.124860004	0.236080006
0.382290006	0.124860004	0.401650012
0.382290006	0.624859989	0.236080006
0.382290006	0.624859989	0.401650012
0.882330000	0.000000000	0.304839998
0.882330000	0.500000000	0.304839998
0.117670000	0.000000000	0.304839998
0.117670000	0.500000000	0.304839998
0.882330000	0.250000000	0.222049996
0.882330000	0.250000000	0.387620002
0.882330000	0.750000000	0.222049996
0.882330000	0.750000000	0.387620002
0.117670000	0.250000000	0.222049996
0.117670000	0.250000000	0.387620002
0.117670000	0.750000000	0.222049996
0.117670000	0.750000000	0.387620002
0.617479980	0.000000000	0.277460009
0.617479980	0.000000000	0.443040013
0.617479980	0.500000000	0.277460009
0.617479980	0.500000000	0.443040013
0.382519990	0.000000000	0.277460009
0.382519990	0.000000000	0.443040013
0.382519990	0.500000000	0.277460009
0.382519990	0.500000000	0.443040013
0.617479980	0.250000000	0.360249996
0.617479980	0.750000000	0.360249996
0.382519990	0.250000000	0.360249996
0.382519990	0.750000000	0.360249996
0.881889999	0.250000000	0.305099994
0.881889999	0.750000000	0.305099994
0.118110001	0.250000000	0.305099994
0.118110001	0.750000000	0.305099994

0.881889999	0.000000000	0.222310007
0.881889999	0.000000000	0.387890011
0.881889999	0.500000000	0.222310007
0.881889999	0.500000000	0.387890011
0.118110001	0.000000000	0.222310007
0.118110001	0.000000000	0.387890011
0.118110001	0.500000000	0.222310007
0.118110001	0.500000000	0.387890011
0.617479980	0.250000000	0.277449995
0.617479980	0.250000000	0.443019986
0.617479980	0.750000000	0.277449995
0.617479980	0.750000000	0.443019986
0.382530004	0.250000000	0.277449995
0.382530004	0.250000000	0.443019986
0.382530004	0.750000000	0.277449995
0.382530004	0.750000000	0.443019986
0.617479980	0.000000000	0.360229999
0.617479980	0.500000000	0.360229999
0.382530004	0.000000000	0.360229999
0.382530004	0.500000000	0.360229999
0.133499995	0.153510004	0.518090010
0.633499980	0.153510004	0.518090010
0.133499995	0.153510004	0.635219991
0.633499980	0.153510004	0.635219991
0.133499995	0.153510004	0.752340019
0.633499980	0.153510004	0.752340019
0.366499990	0.846490026	0.510760009
0.866500020	0.846490026	0.510760009
0.366499990	0.846490026	0.627879977
0.866500020	0.846490026	0.627879977
0.366499990	0.846490026	0.745000005
0.866500020	0.846490026	0.745000005
0.383500010	0.346489996	0.510760009
0.883499980	0.346489996	0.510760009
0.383500010	0.346489996	0.627879977
0.883499980	0.346489996	0.627879977
0.383500010	0.346489996	0.745000005
0.883499980	0.346489996	0.745000005
0.116499998	0.653509974	0.518090010
0.616500020	0.653509974	0.518090010
0.116499998	0.653509974	0.635219991
0.616500020	0.653509974	0.635219991
0.116499998	0.653509974	0.752340019
0.616500020	0.653509974	0.752340019
0.366499990	0.846490026	0.576659977
0.866500020	0.846490026	0.576659977
0.366499990	0.846490026	0.693780005
0.866500020	0.846490026	0.693780005
0.133499995	0.153510004	0.569320023
0.633499980	0.153510004	0.569320023
0.133499995	0.153510004	0.686439991
0.633499980	0.153510004	0.686439991
0.116499998	0.653509974	0.569320023
0.616500020	0.653509974	0.569320023
0.116499998	0.653509974	0.686439991
0.616500020	0.653509974	0.686439991
0.383500010	0.346489996	0.576659977
0.883499980	0.346489996	0.576659977
0.383500010	0.346489996	0.693780005
0.883499980	0.346489996	0.693780005

0.137710005	0.939570010	0.543709993
0.637709975	0.939570010	0.543709993
0.137710005	0.939570010	0.660830021
0.637709975	0.939570010	0.660830021
0.137710005	0.939570010	0.777949989
0.637709975	0.939570010	0.777949989
0.362289995	0.060430001	0.602270007
0.862290025	0.060430001	0.602270007
0.362289995	0.060430001	0.719389975
0.862290025	0.060430001	0.719389975
0.387710005	0.560429990	0.602270007
0.887709975	0.560429990	0.602270007
0.387710005	0.560429990	0.719389975
0.887709975	0.560429990	0.719389975
0.112290002	0.439570010	0.543709993
0.612290025	0.439570010	0.543709993
0.112290002	0.439570010	0.660830021
0.612290025	0.439570010	0.660830021
0.112290002	0.439570010	0.777949989
0.612290025	0.439570010	0.777949989
0.406809986	0.099160001	0.543709993
0.906809986	0.099160001	0.543709993
0.406809986	0.099160001	0.660830021
0.906809986	0.099160001	0.660830021
0.406809986	0.099160001	0.777949989
0.906809986	0.099160001	0.777949989
0.093189999	0.900839984	0.602270007
0.593190014	0.900839984	0.602270007
0.093189999	0.900839984	0.719389975
0.593190014	0.900839984	0.719389975
0.156810001	0.400840014	0.602270007
0.656809986	0.400840014	0.602270007
0.156810001	0.400840014	0.719389975
0.656809986	0.400840014	0.719389975
0.343190014	0.599160016	0.543709993
0.843190014	0.599160016	0.543709993
0.343190014	0.599160016	0.660830021
0.843190014	0.599160016	0.660830021
0.343190014	0.599160016	0.777949989
0.843190014	0.599160016	0.777949989
0.073890001	0.089010000	0.543709993
0.573889971	0.089010000	0.543709993
0.073890001	0.089010000	0.660830021
0.573889971	0.089010000	0.660830021
0.073890001	0.089010000	0.777949989
0.573889971	0.089010000	0.777949989
0.426109999	0.910990000	0.602270007
0.926110029	0.910990000	0.602270007
0.426109999	0.910990000	0.719389975
0.926110029	0.910990000	0.719389975
0.323890001	0.410990000	0.602270007
0.823889971	0.410990000	0.602270007
0.323890001	0.410990000	0.719389975
0.823889971	0.410990000	0.719389975
0.176109999	0.589010000	0.543709993
0.676110029	0.589010000	0.543709993
0.176109999	0.589010000	0.660830021
0.676110029	0.589010000	0.660830021
0.176109999	0.589010000	0.777949989
0.676110029	0.589010000	0.777949989

SI References

1. Padhi AK, Nanjundaswamy KS and Goodenough JB. Phospho-olivines as positive-electrode materials for rechargeable lithium batteries. *J Electrochem Soc* 1997; **144**: 1188–94.
2. Liu C, Neale ZG and Cao G. Understanding electrochemical potentials of cathode materials in rechargeable batteries. *Mater Today* 2016; **19**: 109–23.
3. Gutierrez A, Benedek NA and Manthiram A. Crystal-chemical guide for understanding redox energy variations of $M^{2+/3+}$ couples in polyanion cathodes for lithium-ion batteries. *Chem Mater* 2013; **25**: 4010–6.
4. Manthiram A and Goodenough JB. Lithium insertion into $Fe_2(SO_4)_3$ frameworks. *J Power Sources* 1989; **26**: 403–8.
5. Manthiram A and Goodenough JB. Lithium insertion into $Fe_2(MO_4)_3$ frameworks: comparison of M = W with M = Mo. *J Solid State Chem* 1987; **71**: 349–60.
6. Burns RG. *Mineralogical applications of crystal field theory*: Cambridge university press; 1993.
7. Shi JS, Wu ZJ and Zhou SH *et al.* Dependence of crystal field splitting of 5d levels on hosts in the halide crystals. *Chem Phys Lett* 2003; **380**: 245–50.
8. Burns RG and Sung C-M. The effect of crystal field stabilization on the olivine spinel transition in the system Mg_2SiO_4-Fe_2SiO_4. *Phys Chem Minerals* 1978; **2**: 349–64.
9. Syono Y, Tokonami M and Matsui Y. Crystal field effect on the olivine-spinel transformation. *Phys Earth Planet In* 1971; **4**: 347–52.
10. Jørgensen CK. *Absorption spectra and chemical bonding in complexes*: Elsevier; 2015.
11. Wang Y, Lv J and Zhu L *et al.* Crystal structure prediction *via* particle-swarm optimization. *Phys Rev B* 2010; **82**: 094116.
12. Wang Y, Lv J and Zhu L *et al.* CALYPSO: a method for crystal structure prediction. *Comput Phys Commun* 2012; **183**: 2063–70.
13. Lv J, Wang Y and Zhu L *et al.* Predicted novel high-pressure phases of lithium. *Phys Rev Lett* 2011; **106**: 015503.
14. Zhu L, Wang H and Wang Y *et al.* Substitutional alloy of Bi and Te at high pressure. *Phys Rev Lett* 2011; **106**: 145501.
15. Liu H, Naumov II and Hoffmann R *et al.* Potential high-T_c superconducting lanthanum and yttrium hydrides at high pressure. *Proc Natl Acad Sci USA* 2017; **114**: 6990–5.
16. Simmons G and Wang H. *Single crystal elastic constants and calculated aggregate properties*: The MIT Press; 1971.
17. Yu J, Wang Y and Kong L *et al.* Neuron-mimic smart electrode: a two-dimensional multiscale synergistic strategy for densely packed and high-rate lithium storage. *ACS Nano* 2019; **13**: 9148–60.
18. Wang D, Zhao Y and Lian R *et al.* Atomic insight into the structural transformation and anionic/cationic redox reactions of VS_2 nanosheets in sodium-ion batteries. *J Mater Chem A* 2018; **6**: 15985–92.
19. Yu D, Pang Q and Gao Y *et al.* Hierarchical flower-like VS_2 nanosheets–a high rate-capacity and stable anode material for sodium-ion battery. *Energy Storage Mater* 2018; **11**: 1–7.
20. Bates JB, Dudney NJ and Neudecker BJ *et al.* Preferred orientation of polycrystalline $LiCoO_2$ films. *J Electrochem Soc* 2000; **147**: 59.
21. Garcia-Mendez R, Mizuno F and Zhang R *et al.* Effect of processing conditions of $75Li_2S$-$25P_2S_5$ solid electrolyte on its DC electrochemical behavior. *Electrochim Acta* 2017; **237**: 144–51.
22. Kamaya N, Homma K and Yamakawa Y *et al.* A lithium superionic conductor. *Nat Mater* 2011; **10**: 682–6.
23. Haruyama J, Sodeyama K and Han L *et al.* Space-charge layer effect at interface between oxide cathode and sulfide electrolyte in all-solid-state lithium-ion battery. *Chem Mater* 2014; **26**: 4248–55.
24. Shao-Horn Y, Croguennec L and Delmas C *et al.* Atomic resolution of lithium ions in $LiCoO_2$. *Nat Mater*

2003; **2**: 464–7.

25. Murayama M, Kanno R and Kawamoto Y *et al.* Structure of the thio-LISICON, Li_4GeS_4. *Solid State Ionics* 2002; **154**: 789-94.
26. Kramer D and Ceder G. Tailoring the morphology of $LiCoO_2$: a first principles study. *Chem Mater* 2009; **21**: 3799–809.
27. Jain A, Ong SP and Hautier G *et al.* Commentary: The Materials Project: a materials genome approach to accelerating materials innovation. *APL Mater* 2013; **1**: 011002.
28. Campanella L and Pistoia G. MoO_3: a new electrode material for nonaqueous secondary battery applications. *J Electrochem Soc* 1971; **118**: 1905.
29. Whittingham MS. Electrical energy storage and intercalation chemistry. *Science* 1976; **192**: 1126–7.
30. Bohnke O and Robert G. Electrochemical lithium incorporation into WO_3 and MoO_3 thin films. *Solid State Ionics* 1982; **6**: 115–20.
31. Mulhern PJ and Haering RR. Rechargeable nonaqueous lithium/Mo_6S_8 battery. *Can J Phys* 1984; **62**: 527–31.
32. Julien C, Saikh SI and Nazri GA. Electrochemical studies of disordered MoS_2 as cathode material in lithium batteries. *Mater Sci Eng B* 1992; **15**: 73–7.
33. Zhong Q, Dahn JR and Colbow K. Lithium intercalation into WO_3 and the phase diagram of Li_xWO_3. *Phys Rev B* 1992; **46**: 2554–60.
34. Kumar Sen U, Shaligram A and Mitra S. Intercalation anode material for lithium ion battery based on molybdenum dioxide. *ACS Appl Mater Interfaces* 2014; **6**: 14311–9.
35. Liao Y, Park K-S and Singh P *et al.* Reinvestigation of the electrochemical lithium intercalation in 2H- and 3R-NbS_2. *J Power Sources* 2014; **245**: 27–32.
36. Matsuyama T, Hayashi A and Ozaki T *et al.* Improved electrochemical performance of amorphous TiS_3 electrodes compared to its crystal for all-solid-state rechargeable lithium batteries. *J Ceram Soc Jpn* 2016; **124**: 242–6.
37. Armer CF, Yeoh JS and Li X *et al.* Electrospun vanadium-based oxides as electrode materials. *J Power Sources* 2018; **395**: 414–29.
38. Peng C, Lyu H and Wu L *et al.* Lithium- and magnesium- storage mechanisms of novel hexagonal $NbSe_2$. *ACS Appl Mater Interfaces* 2018; **10**: 36988–95.
39. Wu X, Zou Z and Li S *et al.* Simple synthesis and electrochemical performance of V_6O_{13} cathode materials as lithium-ion batteries. *Ionics* 2019; **25**: 1413–8.
40. Wolfenstine J and Allen J. $LiNiPO_4$-$LiCoPO_4$ solid solutions as cathodes. *J Power Sources* 2004; **136**: 150–3.
41. Melot BC and Tarascon JM. Design and preparation of materials for advanced electrochemical storage. *Accounts Chem Res* 2013; **46**: 1226–38.
42. Winter M, Besenhard JO and Spahr ME *et al.* Insertion electrode materials for rechargeable lithium batteries. *Adv Mater* 1998; **10**: 725–63.
